# Circadian Disruption Exacerbates Innate Immune Responses by Modulating the Bistability of Pro-Inflammatory Signaling: A Dynamical Modeling Study

**DOI:** 10.3390/biomedicines14071454

**Published:** 2026-06-26

**Authors:** Quan Zhou, Qi Ouyang, Hongli Wang

**Affiliations:** 1 The State Key Laboratory for Artificial Microstructures and Mesoscopic Physics, School of Physics, Peking University, Beijing 100871, China; shzhouquan@pku.edu.cn; 2 School of Physics, Zhejiang University, Hangzhou 310058, China; 3 Center for Quantitative Biology, Peking University, Beijing 100871, China

**Keywords:** circadian clock, innate immunity, mathematical model, inflammation, bistability

## Abstract

**Background/Objectives:** Circadian disruption resulting from factors such as jet lag, shift work, or aging leads to exaggerated inflammatory responses and increased disease susceptibility. However, the core dynamical mechanism by which circadian disruption exacerbates innate immune responses remains poorly understood. **Methods:** We develop an integrated mathematical model coupling the mammalian circadian clock with antigen-induced innate immune responses, incorporating key regulatory interactions including glucocorticoid modulation and pro-inflammatory positive feedback loops. **Results:** The model successfully recapitulates experimental data regarding homeostatic immune circadian oscillations and time-dependent gating of acute inflammatory responses. Dynamic analyses reveal that the circadian clock exerts its gating function by modulating the bistable characteristics within pro-inflammatory positive feedback loops. Circadian disruption, simulated as jet lag or age-related reduction in clock gene amplitude, reshapes this bistable landscape and prolongs residence duration in the pathological hyperinflammatory state. **Conclusions:** This shift not only amplifies acute cytokine bursts but also sustains exaggerated inflammatory activity, providing a mechanistic explanation for acute tissue injury and chronic low-grade inflammation (inflammaging) under these circadian disruption scenarios.

## 1. Introduction

The circadian clock is an endogenous timing system that entrains to the day–night cycle, periodically regulating immune function [1,2]. This regulation is mediated by transcription–translation feedback loops (TTFLs) with ~24 h periods, which constitute the core clock mechanism across taxa from fungi to mammals [3] and regulate diverse physiological processes [4]. In the context of immunology in mammals, the circadian gating of innate immunity is particularly critical. The clock modulates bone-marrow homing and peripheral egress by tuning chemokine and receptor expression [5,6,7,8]. In parallel, the circadian system gates the activity of immune cells [9,10], cytokines [11,12], certain receptors [13], and the complement system [14], causing these immune components to oscillate with a near 24 h period. More importantly, cytokine sensitivity to stimulation varies with time of day [11,15], further demonstrating the time dependence of innate immune components under circadian control. Complementing these local mechanisms, the circadian system also exerts systemic control via the hypothalamic–pituitary–adrenal (HPA) axis. The rhythmic secretion of glucocorticoids (CORT), which peaks during the active phase [16], functions mainly as a potent systemic immunomodulatory ‘brake’ that sets the threshold for pro-inflammatory cytokines and coordinates with local molecular clocks [17,18,19].

In recent decades, accumulating evidence has linked circadian abnormality to the pathogenesis of various diseases. Factors such as unhealthy lifestyle factors [20], inflammatory stimuli [21], disrupted light–dark cycle [22], or aging [23] can lead to circadian misalignment or reduced clock gene amplitude, exacerbating various diseases, including Parkinson’s disease [24], depression [25], Alzheimer’s disease [26], and atherosclerosis [27]. Among these, the most direct effect of circadian disruption on the immune system is a significant exaggeration of the host’s innate immune response to external inflammatory stimuli. Animal experiments using mice subjected to serial advances of the light–dark cycle (chronic jet lag, CJL) showed dysregulation of core and peripheral clock genes, leading to altered cytokine release [28,29], and a markedly higher mortality rate after endotoxin challenge (89% vs. 21% in controls) [30]. Studies in rodent shift-work models and workers with chronic shift-work schedules have shown that circadian disruption not only increases the host susceptibility to acute stimuli [31] but also induces persistent low-grade inflammation, as evidenced by significantly elevated concentrations of plasma inflammatory markers and cytokines [32]. Experiments exposing mice to light at night have similarly reported dysregulated cytokine release and responses, as well as anxiety- and depression-like behaviors [25]. Aging is often accompanied by a decrease in the amplitude of core clock gene expression [23], heightening innate responses to stimuli and increasing both acute responses to endotoxin (vs. young mice) and the risk of chronic inflammation [33,34]. Of particular note, extreme circumstances such as sleep deprivation can even induce cytokine storm, leading to uncontrolled elevations of pro-inflammatory cytokines, multiple organ dysfunction syndrome, and even death [35]. Collectively, these studies establish the circadian clock as an inhibitory gatekeeper essential for innate immune homeostasis and provide a foundation for developing integrated dynamic models to dissect its underlying mechanisms.

How circadian dysfunction disrupts this gatekeeper role and drives excessive inflammatory immune reactions is a compelling open question in circadian–immune interaction research. To address such complex, systems-level questions, mathematical modeling has become an indispensable tool. The dynamics of immune responses are increasingly analyzed using complex networks that integrate processes such as genetic regulation, cytokine secretion, and leukocyte trafficking. Foundational work in this area includes mathematical models of the acute inflammatory response to endotoxin challenges, which provided frameworks for understanding the balance between pro- and anti-inflammatory signaling [36,37]. The approach has been extended to infectious diseases. For instance, a recent multicompartment model of host immunity successfully addressed the heterogeneity of COVID-19 clinical presentations by integrating lymphocyte and cytokine trafficking [38]. Furthermore, Zhou et al. introduced a predictive metric termed “immune efficacy” to forecast SARS-CoV-2 outcomes, demonstrating the clinical utility of such models [39]. Building on these advances, researchers have recently focused on modeling the specific influence of circadian rhythms on immunity. For example, sexually dimorphic effects of shift work were investigated using a model of circadian–immune regulation in rats [40], revealing that circadian timing modulates sex-specific cytokine responses. At the systems level, Balit et al. [41] integrated circadian rhythms with CD8^+^ T cell dynamics, finding that a bistable signaling switch enables circadian forcing to generate day–night differences in T cell activation. Most recently, efforts have focused on the time-of-day dependence of viral exposure and vaccination [42]. Critically, these latest studies are beginning to uncover the specific mechanisms hidden in the complex circadian-regulated immunity. They demonstrated that the clock-controlled release of CCL2 in the innate immune response, or the circadian regulation of antigen-presenting cells, directly determines the time-of-day-dependent immune response. These findings suggest that circadian disruption may exacerbate immune responses precisely by perturbing these identified gatekeeping processes.

The purpose of this paper is to construct a comprehensive model of the circadian clock–innate immune response to quantitatively elucidate the core dynamic mechanism by which circadian abnormality regulates exaggerated inflammatory responses. The model for mammals integrates currently known interactions between the circadian clock and innate immunity and considers the effects of two typical circadian disruptors, i.e., jet lag and aging, on innate immune dynamics. Simulations of our model successfully reproduce experimentally observed circadian oscillations and time-dependent inflammatory responses. Dynamic analyses reveal that distorted circadian oscillations induced by jet lag or aging reshape the bistable landscape of a pro-inflammatory positive feedback loop. This reshaping both amplifies acute cytokine responses and prolongs residence time in a high-inflammation state, thereby sustaining exaggerated inflammatory activity. This provides a unifying explanation for chronic low-grade inflammation under abnormal circadian conditions. These mechanistic insights offer a theoretical framework for understanding the origins of chronic inflammation (such as inflammaging) and provide a foundation for the precise control of immune-mediated tissue damage and the development of chronotherapies for diseases associated with circadian rhythm disorders.

## 2. Materials and Methods

### 2.1. Network of Circadian-Regulated Innate Immune Responses

**Figure 1:**
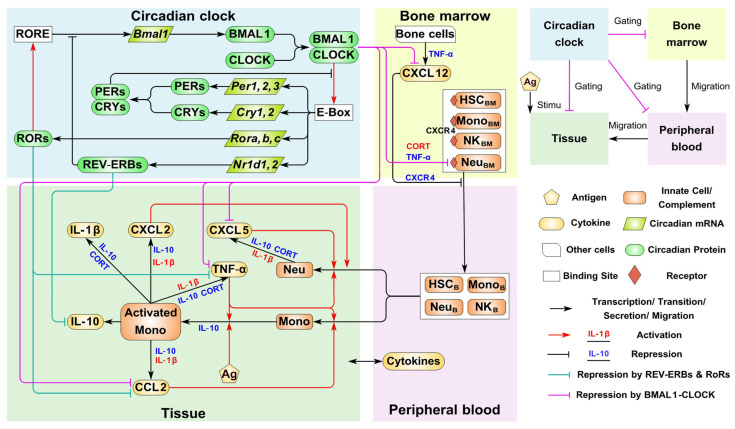
Schematic of the circadian regulation of the innate immune response. Modules are color-coded: light blue represents the canonical TTFL circadian clock, yellow the bone marrow, green the peripheral tissue where innate immunity is activated, and light red the peripheral blood. Circadian clock proteins, including BMAL1, CLOCK, PERs, CRYs, RORs, and REV-ERBs, are depicted as green rounded rectangles. Immune cells, comprising monocytes/macrophages, neutrophils, natural killer cells, and hematopoietic stem cells, are shown as brown rounded rectangles and labeled with the abbreviations Mono, Neu, NK, and HSC, respectively. The immune cells released from the bone marrow circulate in the peripheral blood, where they are recruited or activated by cytokines before entering tissues to participate in the innate immune response. Cytokines are represented by yellow rounded rectangles, with CCL2, IL-1β, TNF-α, IL-10, and CXCL5 exchanged between peripheral tissue and blood. The circadian regulation from the HPA axis is incorporated by introducing the glucocorticoids, which are abbreviated as CORT. The upper-right block summarizes the model architecture, in which the circadian clock module provides regulatory inputs to the bone marrow, peripheral blood, and tissue immune-response modules. In the ODE system, the biological components are represented as dynamic variables, and arrows indicate regulatory or exchange processes among these modules, which are mathematically described in the ODE system.

In mammals, the innate immunity is the first line of fast defense against pathogens and provides fundamental and comprehensive protection. The core components of innate immunity are closely regulated by the circadian clock, particularly in the rhythmic release of innate immune cells from the bone marrow, the rhythmic secretion of cytokines in tissues, and immune response in peripheral tissues to antigen stimulation. Here, we construct a mathematical model of circadian clock-regulated innate immunity, with the aim of quantitatively investigating the effect of clock disruption caused by jet-lag or aging on inflammatory responses due to antigen (such as lipopolysaccharide (LPS)) stimulation. As depicted in Figure 1, the multicompartmental model integrates peripheral tissue, peripheral blood, and bone marrow, which are all regulated by the circadian clock.

The construction of the core circadian clock in our model adheres to the central TTFL molecular mechanism of the mammalian circadian clock (see Figure 1, the module shown in light blue) [3]. In the TTFL, transcription factors BMAL1 and CLOCK first form a heterodimer, binding to E-box elements in the promoter regions of *Per* and *Cry* genes to promote their transcription and translation. Subsequently, PER and CRY proteins form a dimer, enter the nucleus, and inhibit CLOCK–BMAL1 activity by directly binding to the complex, forming the core negative feedback loop. In addition, REV-ERB and ROR proteins constitute secondary feedback loops by binding to the ROR response elements (RORE) of the *Bmal1* gene, respectively, repressing and activating *Bmal1* transcription, thereby stabilizing circadian oscillations. In the circadian clock module, the subtypes of various genes (such as *Per*/*Cry*/*Nr1d*/*Ror*) are lumped together, and the CLOCK protein is assumed to be stably expressed [43]. In our model, the circadian clock is shared across the compartments of peripheral tissue, blood, and bone marrow.

In peripheral tissue, the core mechanisms of acute innate immune response are represented by the compartment in light green in Figure 1 [38]. Upon stimulation by an external antigen (Ag in Figure 1), local monocytes/macrophages (represented with Mono in Figure 1) become activated. The activated monocytes/macrophages rapidly secrete pro-inflammatory cytokines such as Tumor Necrosis Factor-α (TNF-α), interleukin-1β (IL-1β) and C-C motif chemokine ligand 2 (CCL2). TNF-α also reinforces local monocytes/macrophages activation, forming a positive feedback loop that amplifies the inflammatory response. Concurrently, to prevent excessive inflammatory activation, the activated monocytes/macrophages secrete the anti-inflammatory factor Interleukin-10 (IL-10), which constitutes a negative feedback loop by suppressing macrophage activation to reduce cytokine secretion. Additionally, activated monocytes/macrophages secrete the neutrophil-recruiting chemokine C-X-C motif chemokine ligand 2 (CXCL2), which, together with TNF-α, promotes neutrophil (Neu in Figure 1) recruitment from peripheral blood into the stimulated tissue. Neutrophils, after entering the tissue, also secrete C-X-C motif chemokine ligand 5 (CXCL5), which further reinforces neutrophil recruitment in the tissue compartment.

In the peripheral blood compartment (Figure 1, light red module), the cytokines (excluding CXCL2) are represented by a lumped “Cytokines” node. The bidirectional connection between the blood “Cytokines” node and the tissue compartment represents the exchange of these cytokines between blood and tissue. The innate immune cells in blood, such as neutrophils, monocytes, natural killer cells, and hematopoietic stem cells, are released from the bone marrow (Figure 1, light yellow module). The release process is regulated by the CXCL12–CXCR4 axis: CXCL12 in the bone marrow binds to C-X-C chemokine receptor 4 (CXCR4) on immune cells and thereby inhibits their release from the bone marrow [44,45]. This joint retention effect is represented in Figure 1 by an inhibitory link from CXCL12, with CXCR4 indicated as a co-mediating factor. Under inflammatory conditions, this retention effect is weakened, allowing increased release of innate immune cells into the circulation. This is modeled by allowing TNF-α to inhibit both CXCL12 and CXCR4, consistent with the reported TNF-α-dependent reduction in CXCL12 [46]. To simplify the model, the homing process of immune cells is not explicitly considered [47]. Instead, this process is absorbed into the cell loss term, such that the corresponding coefficient represents an effective removal rate rather than a pure intrinsic decay rate.

In Figure 1, the regulation of immunity by the circadian clock is depicted via links connecting clock proteins to target nodes within the immune system. In the tissue compartment, CLOCK–BMAL1 represses the synthesis of CCL2 [12,48], TNF-α [40,49], CXCL5 [17]. RORs inhibit the production of CCL2 [12,48], TNF-α [40,49]. In the bone marrow compartment, CLOCK–BMAL1 inhibits the production of CXCL12 [7] and CXCR4 [6], rhythmically regulating the release of innate immune cells from bone marrow to peripheral blood [8]. To incorporate the circadian regulation by the HPA axis, we also introduced CORT as a key inhibitor in the innate immune system. CORT downregulates the secretion of pro-inflammatory cytokines (TNF-α, IL-1β) and the chemokine CXCL5, reflecting its well-documented immunosuppressive effects [17,18,19]. In addition, CORT is modeled as a positive regulator of CXCR4, thereby reinforcing the retention axis and contributing to circadian control of leukocyte trafficking [50]. Overall, the BMAL1–CLOCK complex serves as a key regulator in this network, yet the inflammatory status is determined by the integrated effects of multiple clock-controlled and CORT-mediated signals. These regulatory pathways enable the system to reproduce the 24 h oscillation of innate immune components and provide a systems-level basis for analyzing the mechanisms of dysregulated immune response under circadian disruption.

### 2.2. Equations

The circadian regulation of innate immune dynamics shown in Figure 1 is described mathematically by a set of coupled nonlinear ordinary differential equations (ODEs) (see Equations (S1)–(S34) and Table S1 for the ODEs and dynamic variables in Supplementary Materials). These ODEs describe key physiological processes within the system—including cell release, transformation, and recruitment; cytokine secretion, diffusion, and degradation; receptor expression and regulation—as well as the cross-regulation among these processes. Together, they constitute a comprehensive dynamic modeling framework for innate immunity under circadian control. Hill functions have been extensively used. For example, if secretion of the cytokine TNF-α is simultaneously inhibited by the anti-inflammatory factor IL-10, this is represented as a modulatory term in the secretion rate of TNF-α of the form $\frac{K_{IL10\text{\_}TNF\alpha }^{2}}{K_{IL10\text{\_}TNF\alpha }^{2}+{\left[IL10\right]}^{2}}$ (see Equation (S27) in Supplementary Materials). For inter-compartmental cell migration processes in our model, such as the recruitment of neutrophils from blood into infected peripheral tissue, cells are recruited by the chemokine CXCL5 and depend on endothelial permeability (modulated by TNF-α), which results in an influx rate proportional to the product of these regulatory terms of the form $\frac{c_{CXCL5\text{\_}Neu}\cdot {\left[CXCL5\right]}^{2}}{K_{CXCL5\text{\_}Neu}^{2}+{\left[CXCL5\right]}^{2}}\cdot \left(1+\frac{h_{TNF\alpha \text{\_}Neu}\cdot \left[TNF\alpha \right]}{K_{TNF\alpha \text{\_}Neu}\;+\left[TNF\alpha \right]}\right)$ (see Equation (S14) in Supplementary Materials). For cytokines diffusing between blood and peripheral tissue, the diffusion rates are determined by the concentration difference between the two sides. Taking IL-10 as an instance (see Equation (S20) in Supplementary Materials), the net inflow to tissue is proportional to $\left[\mathrm{IL}10\mathrm{Blood}\right]/k_{Blood}-\left[\mathrm{IL}10\right]$, and the net inflow to blood is proportional to $\left[\mathrm{IL}10\mathrm{Blood}\right]-k_{Blood}\left[\mathrm{IL}10\right]$, where $k_{Blood}$ is estimated from the volume ratio between peripheral tissue (taking the peritoneal cavity as an example) and blood. The dynamic description for the module of the TTFL circadian clock follows the approach of Wei et al. [43], with key parameters adjusted to capture the experimental observations of circadian clock abnormalities induced by jet lag and aging [22,23]. The diurnal oscillation of CORT at homeostasis is modeled by a non-autonomous ODE with a 24 h sinusoidal driving term (see Equation (S34) in the Supplementary Materials). Upon endotoxin stimulation, the acute elevation of CORT is modeled by introducing an additional time-dependent pulse term into the rate equation for CORT, which fits the experimental data of post-endotoxin serum profiles [51] (see Equation (S34) in Supplementary Materials). The inhibitory effect of CORT on cytokine secretion (such as TNF-α, IL-1β, CXCL5) is represented by Hill-type inhibitory functions (e.g., $\frac{{\;K}_{CORT\text{\_}TNFa}^{n}}{{\;K}_{CORT\text{\_}TNFa}^{n}\;+\;{\left[CORT\right]}^{n}}$) (see Equation (S27) in Supplementary Materials). In our model, the circadian signal that regulates immunity is assumed to originate from peripheral clocks located in tissues, blood, and bone marrow. These peripheral clocks generate a unified circadian rhythmicity that is synchronized by the master clock in the brain’s suprachiasmatic nucleus, under both normal and abnormal conditions such as jet lag and aging. Normal and abnormal circadian rhythmicity are distinguished by the parameter values within the circadian clock module, and the same holds for CORT rhythmicity. Under this simplifying assumption, aging is represented here as clock- and CORT-related rhythm attenuation, rather than intrinsic age-dependent remodeling of the immune system. Accordingly, independently of the circadian clock, the structure and parameters of the immune module, together with the parameters governing clock/CORT-to-immunity coupling, are held constant.

Taken together, the model relies on several key simplifying assumptions. The circadian input to immunity is represented by synchronized peripheral clock signals and CORT dynamics. Jet lag and aging are implemented through parameter changes in the clock and CORT modules; in particular, aging is modeled as clock- and CORT-related rhythm attenuation rather than remodeling of immune function. In addition, cytokine exchange between tissue and blood is modeled as concentration-driven diffusion, immune-cell homing is absorbed into effective loss terms, and the immune-module structure and clock/CORT-to-immunity coupling parameters are kept fixed across conditions. In this paper, the ODEs are solved numerically in MATLAB R2025b (The MathWorks, Inc., Natick, MA, USA) using the stiff solver ode15s, with the parameter values given in the Supplementary Materials.

### 2.3. Parameter Estimation

The ODE model contains 34 state variables and 152 parameters. Of these parameters, 53 are related to the circadian clock, 7 to circadian rhythms of corticosterone (CORT), and 92 to innate immunity. The detailed parameter classifications and numerical values are summarized in Supplementary Tables S2–S6. These parameter values are determined through multiple approaches: inferred from reported experimental measurements, estimated according to physiological ranges, adopted from existing mathematical models, and derived from experimental data fitting. The detailed fitting approach and objective functions are described in the Supplementary Section (S3. Parameter fitting). Parameter determination and fitting adopt a multi-stage strategy, proceeding from the upstream circadian clock to the downstream innate immunity. For the normal circadian clock, the 53 parameters of the upstream clock module are adopted from existing models [40,43] to reproduce the normal circadian rhythm [52] (see Supplementary Table S2). A subset of parameters (Supplementary Table S3) is adjusted to capture the altered circadian rhythms under jet-lagged and aged conditions [22,23]. In parallel, 7 CORT-related parameters (Supplementary Table S4) are fitted to basal CORT profiles under normal, jet-lagged, and aging conditions, together with LPS-induced acute CORT responses [16,53]. Of the 92 innate immune parameters (see Supplementary Tables S5 and S6), 11 are experimentally inferred, 6 are estimated under physiological constraints, and 4 are adopted from existing mathematical models. The remaining 71 innate immune parameters are derived from data fitting. Under unstimulated steady-state conditions, the innate immune system downstream from the circadian clock displays a basal circadian rhythm. As cytokine concentrations remain at low levels with minimal mutual interaction, the components of the system are weakly coupled. This modularity enables a subset of parameters governing circadian rhythms to be fitted largely independently [20,54,55] (Supplementary Table S5). The fitted experimental data include: circadian rhythms in CXCL12 and CXCR4 [7,8]; circadian rhythms of circulating neutrophils, monocytes, NK cells, and HSCs [8]; the coupled rhythms of tissue monocytes and blood CCL2 [20,54]; and finally, the peak-normalized basal TNF-α rhythmic profile [55]. The remaining innate immune parameters are associated with acute immune responses to antigen stimulation, which are derived by fitting to LPS-stimulated experimental datasets (Supplementary Table S6). These parameters are determined in two steps. We first calibrate the core upstream cytokines of TNF-α, IL-1β, and IL-10 using time-of-day-dependent IL-1β and IL-10 data [15] (see Supplementary Figure S2), along with TNF-α, IL-10, and IL-1β measurements under jet-lagged and aged conditions. Relevant datasets are also extracted from those published studies under the normal circadian clock regulation [30,33]. The remaining unresolved parameters associated with downstream CCL2 and CXCL5 responses are estimated using their time-of-day-dependent stimulation data [15].

We carried out sensitivity and identifiability analyses for the 71 fitted parameters associated with innate immunity, with results shown in Supplementary Figures S3–S6 (see also Supplementary S4. Sensitivity Analysis and Parameter Identifiability). Among the top 20 most sensitive parameters, the majority are linked to monocyte (Mono) activation, particularly Ag/AR-related ones, including $K_{Ag\text{\_}AR},c_{Ag\text{\_}AR}$, $K_{IL10\text{\_}Ag}$, $d_{Ag}$, $d_{AR}$, and $d_{Mono}$. As monocyte activation acts upstream of immune responses and controls most cytokine secretion, these parameters are highly sensitive. High-ranking parameters also cover core pro-inflammatory cytokines CCL2 and TNF-α, represented by $c_{CCL2\text{\_}Mono}$, $t_{CCL2}$, $d_{CCL2}$, $K_{CCL2\text{\_}Mono},$ $K_{Mono\text{\_}TNFa}$, $K_{BC\text{\_}TNFa}$, $K_{ROR\text{\_}TNFa}$, and $K_{Mono\text{\_}Il1b}$. Anti-inflammatory IL-10-related parameters $K_{Mono\text{\_}IL10}$, $d_{IL10}$, and $t_{IL10}$ also rank highly, as IL-10 negative feedback closely interacts with monocyte-mediated positive feedback loops and strongly shapes stimulus-induced peak responses. Moreover, $K_{BT}$ mediates cytokine diffusion and trafficking between tissues and blood, and $c_{CXCR4}$ modulates cellular circadian rhythms, explaining their considerable parameter sensitivity. Among the 34 parameters regulating weak oscillations under antigen-free conditions, six are non-identifiable and predominantly associated with CXCL12, including $K_{CXCL12\text{\_}Neu},K_{CXCL12\text{\_}NK}$, and $K_{CXCL12\text{\_}Mono}$. Of the remaining 37 parameters linked to antigen-induced responses, 15 are non-identifiable; these primarily control CXCL5-mediated neutrophil recruitment, such as $c_{Neu\text{\_}CXCL5}$, $c_{CXCL5\text{\_}Neu}$, $K_{CXCL5\text{\_}Neu}$, $h_{IL1b\text{\_}CXCL5}$, $d_{CXCL5}$, $h_{TNFa\text{\_}Neu}$, $K_{TNFa\text{\_}Neu}$ and $d_{Neu}$. These non-identifiable parameters may affect local quantitative predictions, but they are unlikely to dominate the overall inflammatory response patterns, as most parameters were constrained within physiological ranges and the main outputs were further evaluated by sensitivity analysis.

## 3. Results

### 3.1. Homeostatic Circadian Rhythms Under the Regulation of Normal, Jet-Lagged, and Aged Circadian Clocks

**Figure 2:**
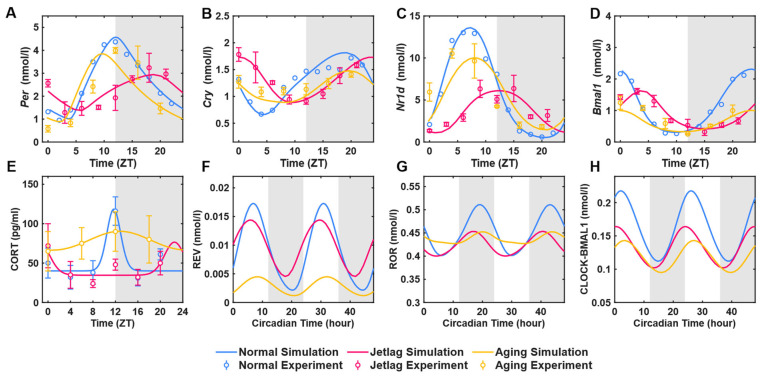
Circadian clock oscillations under normal, jet-lagged, and aged conditions. (A–D) Circadian rhythms in mRNA levels of four core clock genes under normal (blue), jet-lagged (magenta), and aged (yellow) conditions: (**A**) *Per*, (**B**) *Cry*, (**C**) *Nr1d*, and (**D**) *Bmal1*. Lines represent simulation results, and circles denote experimental data [22,23,52]. (**E**) Diurnal oscillations of basal plasma CORT levels under normal, jet-lagged, and aged circadian clocks. The normal rhythm peaks at ZT12, whereas disrupted clocks exhibit dampened amplitudes and altered baselines. Lines represent simulation results, and circles denote experimental data [16,53]. (**F**–**H**) Simulated circadian oscillations in core clock proteins under normal, jet-lagged, and aged conditions over a 48 h period: (**F**) REV-ERB (REV), (**G**) ROR, and (**H**) CLOCK–BMAL1 protein complex. Gray shaded areas indicate the dark phase, corresponding to the active phase in mice.

In our model, the oscillation dynamics of the upstream circadian clock can be simulated independently, as it regulates innate immunity and is not subject to feedback from the downstream innate immune system. Figure 2 depicts the simulation results of normal circadian rhythmicity and disrupted circadian oscillations induced by jet lag and aging, in comparison with experimental data from mice. The mRNA expression levels of four core clock genes, i.e., *Per*, *Cry*, *Nr1d*, and *Bmal1*, are shown in Figure 2A–D. Jet lag induced by a phase-advanced light cycle in mice produces circadian oscillations with a 4–6 h phase shift and decreased oscillation amplitude [22]. In aged mice, the oscillation amplitude is also markedly reduced [23]. While the period of circadian oscillations remains largely unchanged, the mean level of these oscillations is significantly reduced under both jet-lagged and aged conditions compared to that of the normal circadian clock. The simulations agree well with experimental data, accurately capturing the experimentally observed reductions in amplitude and mean value, as well as phase shifts. The comparison between the circadian oscillations of normal circadian clock proteins and the aberrant oscillations induced by shift work and aging is shown in Figure 2F–H. Simulation results for protein levels again demonstrate that both the amplitude and mean level of circadian oscillations are consistently reduced under these circadian disruptions. Compared with the normal clock, the amplitudes of core clock proteins such as PERs, CRYs, and REV-ERBs (Figure 2F), RORs (Figure 2G), and CLOCK–BMAL1 (Figure 2H) are reduced in the disrupted circadian clocks. Notably, the jet-lagged clock exhibits a clear phase shift in the CLOCK–BMAL1 complex, while the aging clock is characterized by a marked suppression of amplitude across all core proteins. These simulated protein-level alterations are consistent with experimentally observed changes under clock disruptions [56,57]. As depicted in Figure 2E, the influence of jet lag and aging on the core circadian clock is also observed in the oscillations of corticosterone in the HPA axis. The simulation of dynamic changes in CORT accurately reproduces the physiological circadian rhythm, characterized by a peak concentration at ZT12 under normal conditions, with a similarly reduced amplitude and mean level in aged and jet-lagged mice, and a delayed phase in the case of jet lag, as observed in experiments [16,53]. Also, the model successfully reproduces the rapid CORT surge following LPS challenge under the normal situation, as in the experiment [51] (see Supplementary Figure S1).

**Figure 3:**
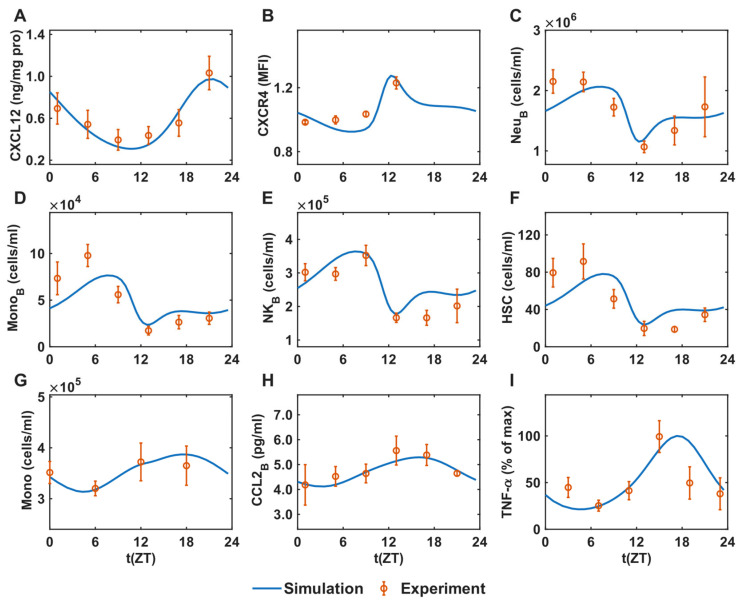
Simulated diurnal oscillations of immune-cell abundance and cytokines regulated by the normal circadian clock under homeostasis without antigen stimulation, compared with experimental data. (**A**) bone marrow CXCL12 levels; (**B**) cell-surface CXCR4 expression; (**C**) peripheral blood neutrophil counts (${Neu}_{B}$); (**D**) peripheral blood monocyte counts (${Mono}_{B}$); (**E**) peripheral blood natural killer cell counts (${NK}_{B}$); (**F**) circulating hematopoietic stem cell counts (HSC); (**G**) tissue monocyte counts; (**H**) plasma CCL2 concentration; and (**I**) tissue TNF-α levels. Blue lines represent simulation results, and orange circles denote experimental data [7,8,20,54,55].

In healthy mammals at homeostasis, the endogenous circadian clock governs robust 24 h rhythms in the abundance and migration of innate immune cells, along with the expression and activity of innate immune receptors, cytokines, and signaling molecules. We next simulated the rhythmicity of innate immunity under homeostatic conditions, regulated by the normal circadian clock oscillations (see Figure 2). Figure 3 depicts the simulated circadian rhythms in the innate immune system, in the absence of infection or other stimuli, in comparison with experimental measurements in mice. The model reproduced the circadian circulation of innate immune cells, showing overall agreement with experimental data [7,8]: circulating neutrophils (Figure 3C), monocytes (Figure 3D), NK cells (Figure 3E), and hematopoietic stem cells (HSC) (Figure 3F) in peripheral blood, peaking around ZT6. CXCL12 concentration in the bone marrow peaks near ZT22 (Figure 3A), exhibiting an antiphase relationship with immune-cell counts in the blood, while cell-surface CXCR4 peaks around ZT12 (Figure 3B). In addition to cellular rhythms, the model’s simulations of oscillations in key immune molecules are consistent with experimental findings: Peripheral tissue monocyte concentration peaks at ZT12–18 (Figure 3G) [20], plasma CCL2 at ZT13–17 (Figure 3H) [54], and tissue TNF-α at ZT15–18 (Figure 3I) [55].

**Figure 4:**
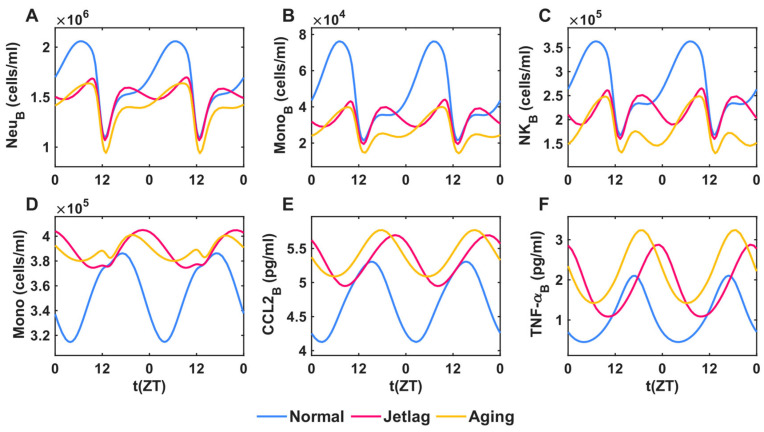
Simulated diurnal oscillations of immune-cell abundance and inflammatory cytokines regulated by circadian clocks that are disrupted by jet lag or aging under homeostasis, in comparison with those under the control of normal circadian clocks. Circadian rhythms are demonstrated for (**A**) neutrophils in blood (${Neu}_{B}$), (**B**) monocytes in blood (${Mono}_{B}$), (**C**) NK cells in blood (${NK}_{B}$), (**D**) monocytes in tissue (Mono), (**E**) CCL2 in blood (${CCL2}_{B}$), and (**F**) TNF-α in blood (${TNFa}_{B}$). Blue, magenta, and yellow lines denote the normal, jet-lagged, and aging conditions, respectively.

We further simulated the homeostatic dynamics of the innate immune system under the regulation of a jet-lagged or aged circadian clock. Figure 4 presents the simulation results of diurnal oscillations in immune cells and inflammatory cytokines without antigen stimulation, in comparison with the circadian oscillations simulated under the regulation of a normal circadian clock. For the circadian rhythms of circulating cell counts (Figure 4A–C), the simulation results show that abnormal circadian clocks attenuate their diurnal oscillation amplitudes. This trend is qualitatively consistent with the findings reported by Zhao et al., who observed disrupted circadian rhythms of circulating leukocytes in mice [58]. For tissue-resident cells (Figure 4D), experimental evidence has shown that tissue monocyte concentrations are elevated in aged mice across multiple peripheral tissues [59], which is also consistent with the simulation results. Finally, for cytokine concentrations (Figure 4E,F), existing studies have demonstrated that mice subjected to chronic jet lag exhibit elevated serum TNF-α levels under unstimulated conditions [60], while immune cells from aged mice show higher expression of inflammatory cytokines without LPS stimulation [59]. These findings are qualitatively consistent with the model-predicted elevation of basal inflammatory levels under abnormal circadian clock conditions.

### 3.2. Exaggerated Acute Inflammatory Responses Under the Regulation of Jet-Lagged and Aged Circadian Clocks

**Figure 5:**
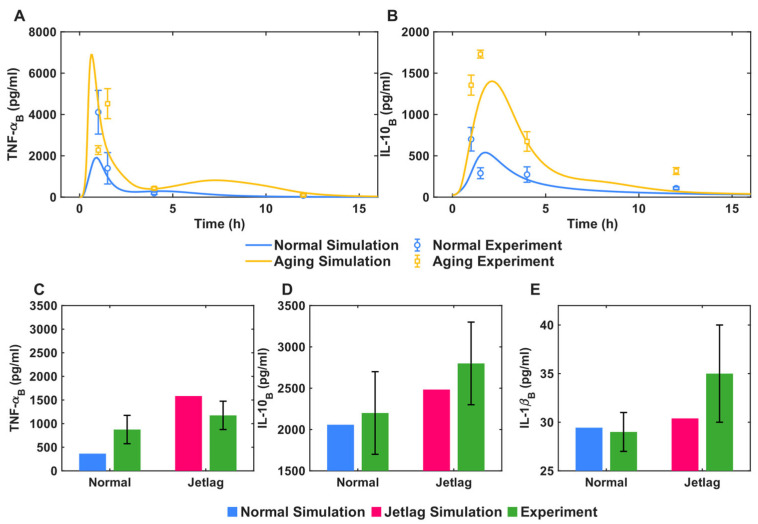
Simulated cytokine responses to LPS stimulation under normal, aged, and jet-lagged circadian conditions compared with experimental data. (**A**,**B**) Time courses of innate-immune cytokines in blood after LPS stimulation (3 mg/kg) at ZT21, regulated by normal (blue) and aged (yellow) clocks: (**A**) TNF-α and (**B**) IL-10. Lines represent simulation results, and symbols denote experimental data [33]. (**C**–**E**) Comparison of acute cytokine responses in blood 1.5 h after LPS stimulation (12.5 mg/kg) at ZT3 between the normal and jet-lagged clocks: (**C**) TNF-α, (**D**) IL-10, and (**E**) IL-1β. Blue bars indicate simulation results for the normal model, magenta bars indicate simulation results for the jet-lagged model, and green bars represent experimental data [30].

We next simulated the dynamic innate immune response in mice challenged with lipopolysaccharide (LPS), with the results presented in Figure 5. Under the same LPS dosage (3 mg/kg), the magnitude of the innate immune response was found to be dependent on the circadian clock. As shown in Figure 5A, under dysregulated circadian control induced by aging [33], the response of TNF-α, a core inflammatory cytokine in the innate immune system, was significantly stronger than that under normal circadian regulation, with higher peak concentrations and prolonged inflammation compared to normal controls, indicating that aged individuals mount a more severe inflammatory response to pathogens. Similarly, Figure 5B illustrates the dynamic response of IL-10, a typical anti-inflammatory cytokine, upon pathogenic stimulation, and shows that its response amplitude in aged mice is also markedly higher than in young mice. The simulation results of Figure 5A,B agree well with the experimental measurements. In mice subjected to jet lag [30], an experimental challenge with lipopolysaccharide (LPS) also elicited a stronger innate immune response than that observed in control mice under normal conditions. We also simulated the innate immune response under dysregulated circadian control induced by jet lag. Figure 5C–E show the blood concentrations of the inflammatory cytokine TNF-α, the pro-inflammatory cytokine interleukin-1β (IL-1β), and the anti-inflammatory cytokine IL-10, simulated at 1.5 h after LPS challenge (12.5 mg/kg), in comparison with experimental measurements [30]. The results demonstrate that the same dose of endotoxin provokes a stronger innate inflammatory response in both aged and jet-lagged mice than in normal mice, which is in general agreement with the experimental findings.

**Figure 6:**
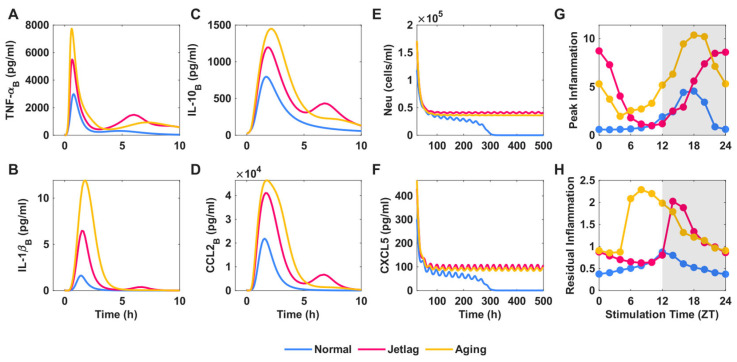
Exaggerated inflammatory responses under the regulation of jet-lagged and aged circadian clocks following LPS stimulation (3 mg/kg). (**A**–**D**) Acute cytokine response curves within 10 h post LPS stimulation at ZT20: (**A**) TNF-α, (**B**) IL-10, (**C**) IL-1β, and (**D**) CCL2. Disrupted circadian rhythms (jet-lagged and aged circadian clock) exhibit markedly higher peak cytokine levels relative to the normal circadian rhythm (blue). (**E**,**F**) Long-term resolution dynamics of neutrophil counts (Neu) and CXCL5 concentrations over 500 h post-stimulation. (**G**,**H**) The peak and residual inflammation coefficients, defined in Equations (1) and (2), are plotted as functions of LPS stimulation time, revealing that disrupted circadian regulation exhibits persistent elevation indicative of chronic inflammation. Gray shaded areas indicate the dark phase. In panels (**G**,**H**), circles indicate discrete LPS stimulation time points.

A more direct comparison of simulated innate immune responses under the regulation of normal, jet-lagged, and aged circadian clocks is demonstrated in Figure 6A–D. Under the same dose of LPS stimulation (3 mg/kg), the dynamic responses of cytokines TNF-α, IL-10, IL-1β, and CCL2 within 10 h after LPS stimulation were strongest under the regulation of the aged circadian clock, followed by those under the jet-lagged circadian clock. Inflammatory cytokine responses under both conditions exhibited greater intensity and longer duration than those under normal circadian control, with particularly pronounced differences in peak levels. Figure 6E,F show neutrophil and CXCL5 dynamics over an extended 500 h time course after LPS stimulation, further demonstrating that the innate immune response under disrupted circadian clock regulation exhibits a stronger inflammatory response compared to normal conditions and incurs a chronic low-grade inflammation [61].

Figure 6A–F showed the results of LPS stimulation at a specific time of day (ZT20). To delineate inflammatory responses for LPS applied at different times across the day and comprehensively compare inflammatory intensity under normal and disrupted circadian regulation, we systematically simulated innate immune responses in mice receiving an identical LPS dose (3 mg/kg) at 12 time points across the day (i.e., $t_{i}$, *i* = 1, 2,…,12 for ZT2, ZT4, …,ZT24). For the LPS challenge at time $t_{i}$, we determined from the simulated innate immune response the peak cytokine concentration $P_{j,k}(t_{i})$, where *j* denotes one of the cytokines (TNF-α, IL-10, IL-1β, CCL2, or CXCL5), and *k* corresponds to the normal, jet-lagged, or aged circadian clock regulation. Similarly, the residual inflammation can be quantified by the cytokine level $R_{j,k}(t_{i})$ at 12 h after LPS challenge at time $t_{i}$. In terms of $P_{j,k}(t_{i})$ and $R_{j,k}(t_{i})$, a peak inflammation coefficient (PIC) and a residual inflammation coefficient (RIC) can be defined to quantitatively evaluate the magnitude and persistence of inflammatory responses, both of which depend on the LPS stimulation time $t_{i}$ and the normality or dysregulation of circadian rhythms (subscript-k-dependent),

(1) $${PIC}_{k}(t_{i})=\sum_{j}\frac{P_{j,k}(t_{i})}{\mathrm{Max}\left\{P_{j,k}\left(t_{i}\right),i=1,2,\ldots 12,k=normal\right\}},$$

(2) $${RIC}_{k}(t_{i})=\sum_{j}\frac{R_{j,k}(t_{i})}{\mathrm{Max}\left\{R_{j,k}\left(t_{i}\right),i=1,2,\ldots 12,k=normal\right\}},$$

where the coefficients are normalized, respectively, to the maximum values of $P_{j,k}(t_{i})$ and $R_{j,k}(t_{i})$ simulated under normal circadian regulation. Figure 6G–H depicts PIC and RIC as functions of LPS stimulation time. The results reveal that the magnitude and persistence of acute inflammatory burden under the regulation of jet-lagged and aged circadian clocks are systematically higher than those under normal circadian regulation. Together, the increased residual inflammatory score and the persistent CXCL5 elevation are consistent with reduced inflammation resolution and the emergence of chronic low-grade inflammation [61].

### 3.3. Mechanism of Exaggerated Acute Inflammation Induced by Circadian Disruption

The mechanism of exaggerated inflammation induced by jet-lagged and aged circadian clocks is rooted in the inflammatory response that is regulated by core clock proteins such as BMAL1–CLOCK, RORs, and CORT. In innate immunity, inflammatory responses involving cytokines TNF-α, IL-1β, IL-10, etc., are closely related to the monocyte–TNF-α positive feedback loop. Upon activation, monocytes rapidly secrete the pro-inflammatory cytokines TNF-α and IL-1β. TNF-α facilitates monocyte recruitment from the circulation into peripheral tissues and further promotes their tissue-level activation, whereas IL-1β released by activated monocytes in turn augments TNF-α production, thereby forming a self-amplifying positive feedback loop of inflammation. The feedback circuit is under circadian regulation via clock proteins: the CLOCK–BMAL1 complex suppresses TNF-α synthesis, ROR family proteins constrain TNF-α production, and the immunosuppressive hormone CORT also downregulates TNF-α secretion. To explore the dynamic mechanism, we extracted the ODEs governing monocytes and TNF-α for separate analysis, which are presented in the following form (see Supplementary Equations (S16) and (S27)),

(3) $$\frac{d\left[Mono\right]}{dt}=-d_{Mono}\cdot \left[Mono\right]+B_{Mono}\left(1+\frac{h_{TNFa\text{\_}Mono}\cdot {\left[TNFa\right]}^{3}}{K_{TNFa\text{\_}Mono}^{3}+{\left[TNFa\right]}^{3}}\right),$$

(4) $$\frac{d\left[TNFa\right]}{dt}=-{d^{\prime }}_{TNFa}\cdot \left[TNFa\right]+C_{TNFa}\cdot \frac{{\left(\left[Mono\right]\cdot AR\right)}^{4}}{{\left(\left[Mono\right]\cdot AR\right)}^{4}+K_{{TNFa}_{Mono}}^{4}},$$

where ${d^{\prime }}_{TNFa}$ is the effective decay rate, which has merged the decay of TNF-α and its diffusion into the circulation. Equations (3) and (4) are non-autonomous, which depend on the dynamic variable AR (i.e., the fraction of activated monocytes in tissue) and time-dependent parameters $B_{Mono}$ and $C_{TNFa}$ defined by,

(5) $$B_{Mono}\equiv c_{CCL2\text{\_}Mono}\cdot \frac{{\left[MonoBlood\right]}^{2}}{{\left[MonoBlood\right]}^{2}+K_{BT\text{\_}Mono}^{2}}\cdot \frac{{\left[CCL2\right]}^{4}}{K_{CCL2\text{\_}Mono}^{4}+{\left[CCL2\right]}^{4}},$$

(6) $$\begin{array}{cc} C_{TNFa} & \equiv c_{Mono\text{\_}TNFa}\cdot \frac{K_{IL10\text{\_}TNFa}^{3}}{K_{IL10\text{\_}TNFa}^{3}+{\left[IL10\right]}^{3}}\cdot \left(1+h_{IL1b\text{\_}TNFa}\cdot \frac{{\left[IL1b\right]}^{3}}{K_{IL1b\text{\_}TNFa}^{3}+{\left[IL1b\right]}^{3}}\right)\\ & \cdot \frac{K_{ROR\text{\_}TNFa}^{4}}{K_{ROR\text{\_}TNFa}^{4}+{\left[ROR\right]}^{4}}\cdot \frac{K_{BC\text{\_}TNFa}^{3}}{K_{BC\text{\_}TNFa}^{3}+{\left[BMAL1-CLOCK\right]}^{3}}\cdot \frac{K_{\mathrm{CORT}\text{\_}\mathrm{TNFa}}^{2}}{K_{\mathrm{CORT}\text{\_}\mathrm{TNFa}}^{2}+{\left[\mathrm{CORT}\right]}^{2}}. \end{array}$$

**Figure 7:**
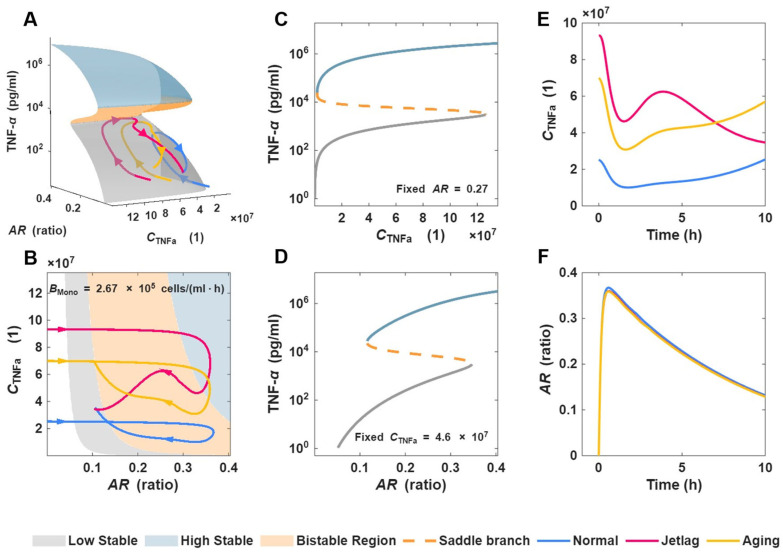
Bifurcation analysis of the two-variable system described by Equations (3) and (4), with AR and $C_{TNFa}$ taken as control parameters. (**A**) Steady-state surface as a function of AR and $C_{TNFa}$, along with the trajectories of (AR, $C_{TNFa}$, TNF-α) under normal, jet-lagged, and aged circadian rhythms. (**B**) Projections of the steady-state surface and the three trajectories in panel (**A**) onto the AR-$C_{TNFa}$ plane, showing monostable and bistable regions as well as the trajectory evolution across these regimes. (**C**,**D**) Two-dimensional cross-sections of the bifurcation surface at fixed AR = 0.27 (**C**) and fixed $C_{TNFa}=4.5\times 10^{7}$ (**D**), illustrating saddle-node bifurcations and bistability characteristics. (**E**,**F**) Time courses of $C_{TNFa}$ (**E**) and AR (**F**) under normal, jet-lagged, and aged circadian rhythms. The trajectories are generated with an LPS dose of 2 mg/kg administered at ZT0. Arrows indicate the direction of trajectory evolution, and dashed orange curves in panels (**C**,**D**) indicate unstable saddle branches.

$B_{Mono}$ and $C_{TNFa}$ represent the recruitment rate of monocytes from the bloodstream and the secretion rate of TNF-α, respectively. Simulations indicate that $B_{Mono}$ varies little over time and is unaffected by the circadian clock. In the subsequent analysis, $B_{Mono}$ is set as a fixed parameter, whereas AR and $C_{TNFa}$ serve as control parameters. In Equation (6), $C_{TNFa}$ is dynamically regulated by the circadian clock through BMAL1–CLOCK, RORs, and CORT. Figure 7A depicts the bifurcation diagram for Equations (3) and (4), illustrating how the steady state varies with AR and $C_{TNFa}$ and revealing a folded bistable surface. Figure 7A also shows the trajectories of (AR, $C_{TNFa}$, TNF-α) under the regulation of normal, jet-lagged, and aged circadian rhythms. Figure 7B presents the projections of the steady-state surface in Figure 7A, together with the three trajectories onto the $AR-C_{TNFa}$ parameter plane. The two trajectories corresponding to aberrant circadian rhythms cross the bistable regime and invade the high TNF-α state, which corresponds to the high inflammation state. Figure 7C,D display cross-sections of the steady-state surface in Figure 7 at fixed values of $C_{TNFa}$ and AR, clearly illustrating the saddle-node bifurcation in Equations (3) and (4). Figure 7E,F show the time courses of $C_{TNFa}$ and AR, which act as control parameters in the two-variable dynamical system of Equations (3) and (4). Notably, $C_{TNFa}$ exhibits strong dependence on circadian rhythm, with its variation range differing markedly between normal and the two aberrant circadian conditions. AR exhibits negligible dependence on circadian clock types but undergoes activation and subsequent decline upon LPS stimulation. In Figure 7E, $C_{TNFa}$ ranges from $1\times 10^{7}$ to $4\times 10^{7}$, remaining confined to the low-inflammatory state in the bistable regime and failing to cross the right-side saddle-node point in Figure 7C to access the hyperinflammatory state. In contrast, under jet-lagged and aged circadian clock regulation, $C_{TNFa}$ exceeds this saddle-node point, enabling the system to dynamically transition into the hyperinflammatory state. This observation is consistent with the trajectory shown in Figure 7B.

**Figure 8:**
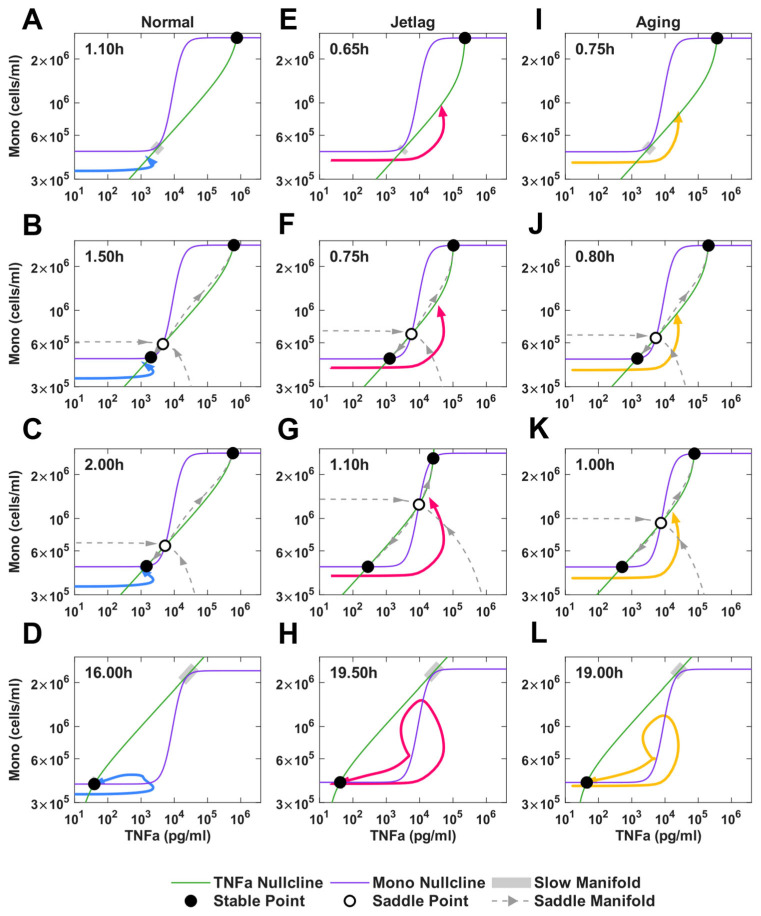
Time-resolved snapshots of trajectories in the monocyte–TNF-α phase plane following LPS stimulation (3 mg/kg) at ZT0. Columns show trajectories under normal (**A**–**D**), jet-lagged (**E**–**H**), and aged (**I**–**L**) circadian clock regulations. The system exhibits a prolonged residence in the hyperinflammatory region under jet-lagged and aged circadian clock conditions. Blue, magenta, and yellow trajectories indicate normal, jet-lagged, and aged circadian conditions, respectively; green and purple curves indicate the TNF-α and Mono nullclines, respectively.

The mechanism of enhanced inflammation induced by circadian disruption shown in Figure 7 can be further elucidated by the trajectories on monocyte–TNF-α phase plane in Figure 8. The transient trajectories under the regulation of normal and two aberrant circadian clocks are simulated with an endotoxin challenge (3 mg/kg LPS). In Figure 8, the “S”-shaped monocyte nullcline (d[Mono]/dt = 0) remains fixed, whereas the nearly straight TNF-α nullcline (d[TNFa]/dt = 0) shifts upper-left with variations in AR and $C_{TNFa}$. During this process, the number of intersection points between the two nullclines transiently changes from one to two and eventually returns to one via saddle-node bifurcation. Initially, the intersection of the two nullclines gives rise to a single high steady-state fixed point (high-inflammation), which attracts system trajectories toward itself, eliciting an acute inflammatory response. As the TNF-α nullcline shifts, a low steady-state fixed point gradually emerges to ensure a self-limiting inflammatory response, while the high steady-state fixed point is annihilated thereafter. Correspondingly, the system trajectory rises to a peak and then declines, eventually being attracted and settling into the low monostable steady state (low-inflammation). Under normal circadian clock regulation (Figure 8A–D), the trajectory remains confined within the low-inflammatory region. By contrast, under jet-lagged (Figure 8E–H) and aged (Figure 8I–L) circadian clock regulation, the trajectory departs substantially from the low-inflammatory region, crosses the saddle point between the two bistable states transiently, and moves toward the high-state fixed point. Although the trajectory eventually converges to the low-state fixed point in all three cases, it sustains a prolonged residence within the hyperinflammatory region under aberrant circadian conditions. These analyses uncover a dynamic mechanism underlying how disrupted circadian clocks drive exaggerated acute inflammatory responses.

**Figure 9:**
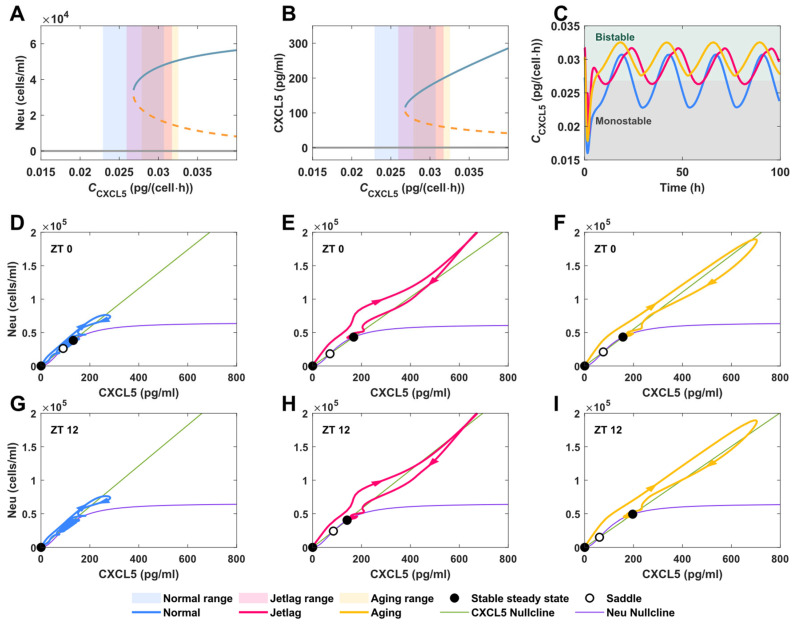
Neutrophil–CXCL5 positive feedback-mediated bistability (**A**–**C**) and circadian clock-regulated dynamics under normal (**D**,**G**), jet-lagged (**E**,**H**), and aged (**F**,**I**) conditions after prolonged antigen stimulation. The trajectories in panels (**D**–**I**) represent simulation results of LPS stimulation at a dose of 3 mg/kg: panels (**D**–**F**) correspond to ZT0 stimulation, and panels (**G**–**I**) to ZT12 stimulation. The bifurcation diagrams (**A**,**B**) are obtained based on the isolated 2-variable ODEs for neutrophil and CXCL5 (see Supplementary Equations (S35) and (S36)), where $C_{CXCL5}$ is the control parameter influenced by the circadian clock. In panels (**A**,**B**), solid curves indicate stable steady-state branches, whereas dashed orange curves indicate unstable branches associated with saddle points.

Long after antigen challenge, most variables in the system decline to very low levels, while a small number remain at relatively high magnitudes. Persistent higher levels in neutrophil and CXCL5, shown in Figure 6E,F, suggest a chronic low-grade inflammation that disrupts circadian clocks. As we have checked, the dynamic origin for the emergence of chronic low-grade inflammation lies in the positive feedback loop in which neutrophils secrete the chemokine CXCL5 that enhances their migration from blood to tissue. The positive feedback loop is regulated by the circadian clock, in which the production of CXCL5 is repressed by BMAL1–CLOCK. Saddle-node bifurcation and bistability occur in the two-variable neutrophils–CXCL5 ODEs (see Supplementary Equations (S35) and (S36)) as illustrated in Figure 9. It depicts that the fluctuation range under a normal circadian clock spans both the “single low state” region and the “bistability” region, enabling the system to gradually return to the low state after stimulation, whereas the fluctuation ranges under disrupted circadian clocks cover mainly the “bistability” region, so once the system is activated into the high state, it ultimately stabilizes in the high-inflammation state. This explains why circadian disruption can induce chronic inflammation.

## 4. Discussion

The circadian clock significantly influences innate immune processes, and its disruption impairs immune responses. Immune dysregulation can trigger acute cytokine storms and chronic inflammation (e.g., inflammaging), which can culminate in immune-mediated inflammatory diseases (IMIDs), including autoimmune disorders. While these effects are well-documented experimentally, the underlying quantitative dynamic mechanisms remain incompletely understood. Here, we proposed a comprehensive model that integrates currently known interactions through which the circadian clock modulates innate immunity. This model encompasses the regulatory roles of both the canonical TTFL circadian machinery and rhythmic CORT signaling within the HPA axis. We further explore the dynamic mechanisms by which circadian disruption drives exaggerated inflammatory responses: the circadian clock modulates bistability arising from pro-inflammatory feedback loops, thereby governing residence times in low- and high-inflammation states, and shaping the magnitude and duration of inflammatory responses. With properly calibrated parameters, the model recapitulates homeostatic circadian oscillations and exaggerated acute inflammatory responses to antigen challenge under normal, jet-lagged, and aging circadian conditions, consistent with experimental observations. This framework provides a mechanistic dynamic interpretation of how circadian disruption exacerbates acute inflammatory injury and may contribute to chronic inflammatory phenotypes under the modeled disruption scenarios, filling a critical knowledge gap in prior qualitative studies.

The uncovered mechanism sheds light on immune dysregulation linked to diverse circadian rhythm disorders and underscores the therapeutic potential of circadian-targeted strategies. Multiple clock-targeted interventions have been reported to ameliorate immune dysregulation [62]. For example, melatonin—a key circadian hormone—can reduce neutrophil infiltration and the release of pro-inflammatory cytokines during acute inflammation, thereby limiting tissue injury [63]. In mice, REV-ERBα agonists strengthen circadian oscillations and attenuate adaptive-immune overactivation, reducing disease severity in experimental autoimmune encephalomyelitis (EAE) and inflammatory bowel diseases (IBD) [64]. More recently, the plant-derived compound jujuboside A (JuA) has been shown to modulate (e.g., inhibit) NF-κB signaling, thereby enhancing clock-linked anti-inflammatory effects and reducing dysregulated production of inflammatory cytokines [65]. Guided by our model’s insights, we suggest that these interventions may confer therapeutic benefits by: (1) restoring robust, physiological-amplitude circadian oscillations within innate immune pathways; (2) enhancing system robustness to perturbations (i.e., enlarging the basin of attraction of the low-inflammation state); and (3) raising the switching threshold, thereby reducing transitions to the high-inflammation state. In the future, by combining this model with disease-specific characteristics, one can design targeted chronotherapies to prevent and treat damage caused by excessive immune responses. Future experimental studies may examine whether, under defined inflammatory stimulation, different forms or degrees of circadian disruption produce threshold-like increases in inflammatory amplitude. Finally, our framework provides a scalable foundation for exploring the circadian regulation of adaptive immunity and behavior.

Several limitations of this study should be noted. First, our model adopts a framework of unidirectional regulation (from the clock to immunity), but in recent years, several experiments have reported feedback effects of inflammation on the clock that lead to circadian disruption [66]. For example, the NF-κB pathway, which is activated at the same time as inflammation, upregulates miR-155. This microRNA directly targets and degrades *Bmal1* mRNA, disturbing the core clock loop [67]. Meanwhile, pro-inflammatory cytokines can also affect the clock: TNF-α can inhibit the expression of clock genes by interfering with E-box-mediated transcription [68]; IL-1β can enhance the degradation efficiency of REV-ERB protein via the ubiquitin–proteasome pathway, thereby suppressing its concentration [69]. Some models have already taken related aspects into account by adding pathways through which inflammatory cytokines feed back onto clock proteins [40,70]. Second, as our current framework focuses on the acute-phase kinetics of inflammation, it does not explicitly model tissue injury or the subsequent liberation of damage-associated molecular patterns (DAMPs). These secondary processes often trigger more severe cytokine storms and typically manifest on a timescale extending beyond the immediate acute response modeled here. In this context, the exaggerated short-term inflammatory bursts predicted by our model serve as a precursor indicator for such pathological risks; an intensified acute response implies a higher probability of crossing the threshold into uncontrolled systemic damage [71]. Third, aging-associated immune dysfunction is much broader than the reduction in clock-gene amplitude captured in our model. We considered only the effects of aging-related attenuation of circadian rhythms, rather than providing a complete representation of immune aging. We deliberately simplified aging to weaken circadian and CORT rhythms in order to isolate the causal contribution of this specific axis. Thus, rhythmic decline is a sufficient, but not necessary, condition for bistability-mediated proinflammation. In real aging, additional factors independent of circadian regulation (e.g., intrinsic immune remodeling, altered hematopoiesis, inflammation-related pathways, and changes in immune cell function) [59,72] would likely contribute to chronic low-grade inflammation and exacerbate this pro-inflammatory response. The predictions of our model should therefore be viewed as conservative, and restoring rhythms alone may be an overestimated therapeutic strategy. Our model provides primarily mechanistic insight into the effect of aging-associated circadian dampening. A more complete model that incorporates these additional factors would be valuable in the future. Our model focuses only on two types of circadian disruption—jet lag and aging—whereas in practice, different rhythm disorders (e.g., repeated phase shifts in shift work, circadian desynchrony among organs [30]) may have differential effects on immunity. Moreover, our simulations approximate circadian disruption as a chronic, system-wide alteration of clock amplitude or total phase, without considering transient or tissue-specific internal desynchronization. Future work could construct more fine-grained models of circadian disruptions and incorporate individual-specific parameter estimation with quantitative metrics that jointly assess host-protection efficacy and immunopathology, enabling comparison of immune outcomes across different forms of circadian disruption. This may also help reveal how the immune system has evolved trade-offs between costs and benefits during evolution to ultimately form its current complex architecture—strong and powerful enough for defense, yet without incurring excessive immune damage too frequently [73,74]. This perspective may also inform future modeling of immune tolerance and autoimmune diseases. Furthermore, our representation of neuroendocrine regulation remains simplified. By modeling CORT dynamics solely through empirical functions, we treated the HPA axis as the exclusive regulatory pathway, neglecting the critical role of the sympathetic nervous system (SNS). Crucially, catecholamines regulate leukocyte trafficking via mechanisms distinct from glucocorticoids. For instance, catecholamines trigger rapid mobilization of leukocytes from the marginal pool during acute stress [75]. Consequently, the absence of adrenergic variables prevents our model from capturing the dynamics of early immune mobilization. Future comprehensive models should integrate these signaling pathways to fully reflect stress physiology.

## 5. Conclusions

We developed a mechanistic, multi-compartment mathematical model linking innate immunity to distinct circadian clocks (normal or disturbed). The model quantitatively reproduces daily rhythms in leukocyte trafficking and cytokine secretion, as well as acute immune responses to endotoxin challenge under the regulation of the normal and disrupted clocks induced by jet lag or aging. The results indicate that the framework captures key features of circadian gating of innate immune responses at both the molecular and systems levels. The model provides a compact dynamics view of how clock disruption can shift innate immunity toward more persistent and exaggerated inflammatory responses. Instead of merely scaling cytokine levels, circadian disruption alters core inflammatory circuits, making the system more prone to high and sustained activation following transient stimuli. This perspective offers a mechanistic interpretation of how these modeled forms of circadian disruption—specifically jet-lag and aging-associated amplitude reduction—can promote dysregulated inflammation. More broadly, our framework provides a quantitative foundation for virtual chronotherapy simulations. By systematically modulating circadian perturbations or integrating disease- and tissue-specific modules into the current model, future work could explore how clock-targeted or time-of-day-based interventions prevent and mitigate immune-mediated tissue damage. Ultimately, our findings highlight that sustaining robust circadian rhythms constitutes a fundamental strategy to keep innate immune responses protective yet self-limiting, thereby maintaining systemic physiological homeostasis.

## Data Availability

The original contributions presented in this study are included in the article/Supplementary Material. Further inquiries can be directed to the corresponding author.

## Supplementary Materials

The following supporting information can be downloaded at https://www.mdpi.com/article/10.3390/biomedicines14071454/s1, supplementary figures, mathematical model equations, parameter fitting, sensitivity analysis, parameter identifiability, and parameter tables, including Figure S1: Simulated acute plasma corticosterone responses following LPS administration; Figure S2: Simulated and experimental cytokine responses to LPS stimulation at various circadian times; Figure S3: Parameter sensitivity ranking of all 92 parameters; Figure S4: F-normalized profile-based identifiability diagnostics for 71 fitted innate-immune parameters; Figure S5: Task-specific SRCC sensitivity screening of fitted immune parameters; Figure S6: FIM-based local collinearity analysis; Table S1: List of variables; Table S2: Parameters of the normal circadian clock; Table S3: Parameters modified in disrupted circadian models; Table S4: Parameter values of CORT regulation under different circadian conditions; Table S5: Parameters of basal cell-trafficking rhythms and stimulation-independent secretion; Table S6: Parameters of stimulated inflammatory responses. Additional references used only in the Supplementary Materials are included in the reference list [76,77,78,79,80,81,82,83,84,85,86,87,88,89,90,91,92,93,94,95,96,97,98,99,100].
