# Supplementary material for: Circadian Disruption Exacerbates Innate Immune Responses by Modulating the Bistability of Pro-Inflammatory Signaling: A Dynamical Modeling Study
Source: Biomedicines. 2026 Jun 26;14(7):1454. doi: 10.3390/biomedicines14071454 (PMC13406095; doi:10.3390/biomedicines14071454)
Supplement: Supplementary file 1 [file biomedicines-14-01454-s001.zip › biomedicines-4363829-supplementary.pdf]

Supplementary Materials for

Circadian disruption exacerbates innate immune responses by modulating the bistability of pro-inflammatory signaling: A dynamical modeling study

Authors: Quan Zhou, Qi Ouyang, and Hongli Wang

Content

S1. Supplementary Figures 2
S2. Mathematical Model Equations 5
S2.1. Model variables 5
Table S1. List of variables. 5
S2.2. Equations of the mathematical model 6
Equation S1–S12. Circadian mRNAs and proteins 6
Equation S13–S19. Immune cells and receptors 7
Equation S20–S34. Immune cytokines, activation state, antigen, and CORT 8
Equation S35–S36. Isolated two-variable ODEs for the neutrophil–CXCL5 positive feedback loop 10
S3. Parameter fitting 10
S3.1. Calibration of Disrupted Circadian Clocks and CORT Dynamics 11
S3.2. Fitting Basal Cell-Trafficking Rhythms and Stimulation-Independent Secretion 11
S3.3. Fitting Stimulated Inflammatory Responses 12
S4. Sensitivity Analysis and Parameter Identifiability 13
S4.1. Local perturbation sensitivity analysis 13
S4.2. F-normalized profile-based identifiability diagnostics 13
S4.3. SRCC sensitivity screening and FIM-based auxiliary interpretation 15
S5. Supplementary Parameter Tables 15
Table S2. Parameters of the normal circadian clock. 16
Table S3. Parameters modified in disrupted circadian models. 17
Table S4. Parameter values of CORT regulation under different circadian conditions. 18
Table S5. Parameters of basal cell-trafficking rhythms and stimulation-independent secretion. 18
Table S6. Parameters of stimulated inflammatory responses. 21
Supplementary References 24

S1. Supplementary Figures

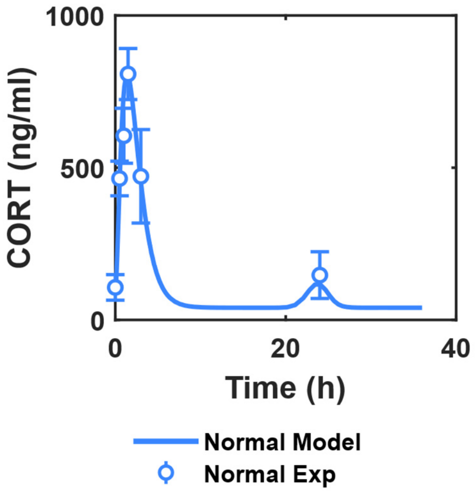

Figure S1. Simulated acute plasma corticosterone (CORT) responses were compared with experimental data [1] following 0.5 mg/kg LPS administration at ZT12. The minor secondary peak near 24 h indicates the recurrence of endogenous basal CORT fluctuations after the abatement of acute inflammatory responses.

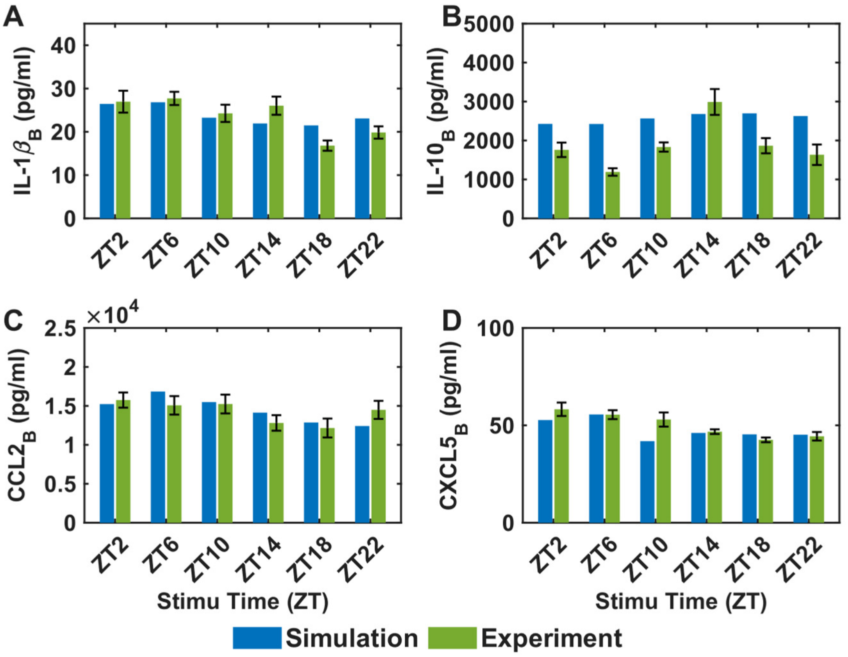

Figure S2. Simulated and experimental cytokine responses to LPS stimulation at various circadian times under normal circadian control. (A–D) Blood cytokine concentrations at 2 h after LPS exposure at ZT2, ZT6, ZT10, ZT14, ZT18 and ZT22: (A) $\mathrm{IL1b}_B$, (B) $\mathrm{IL10}_B$, (C) $\mathrm{CCL2}_B$, (D) $\mathrm{CXCL5}_B$. Blue bars represent simulated results, green bars represent experimental data [2]. Error bars indicate data variability.

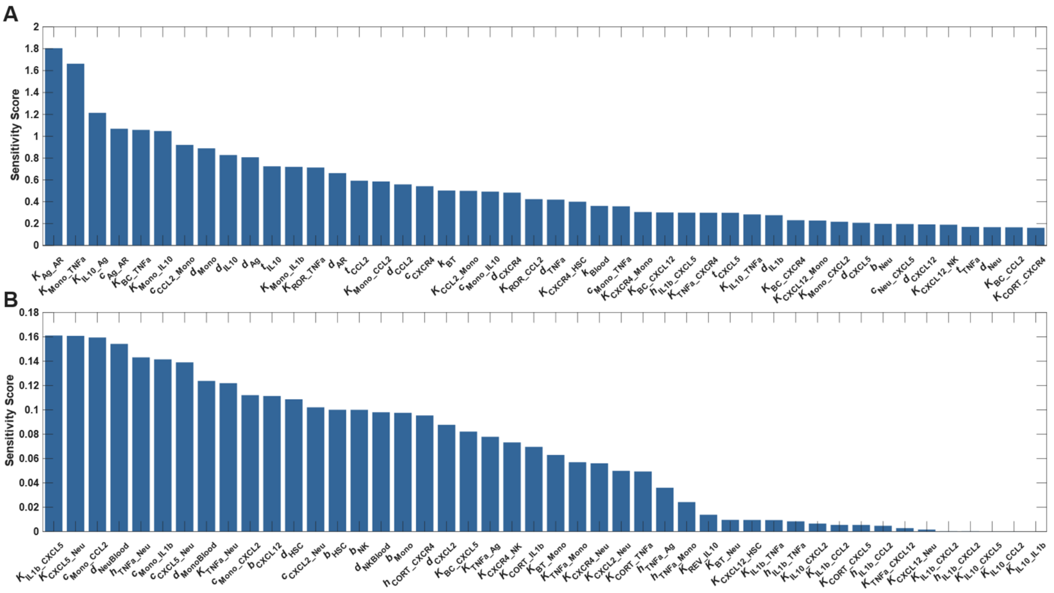

Figure S3. Parameter sensitivity ranking of all 92 parameters for innate immune responses. (A) Top-ranked high-sensitivity parameters; (B) other low-sensitivity parameters. The sensitivity of parameter *k* was quantified by examining the impacts of a 5% increase and decrease in *k* on the simulated results.

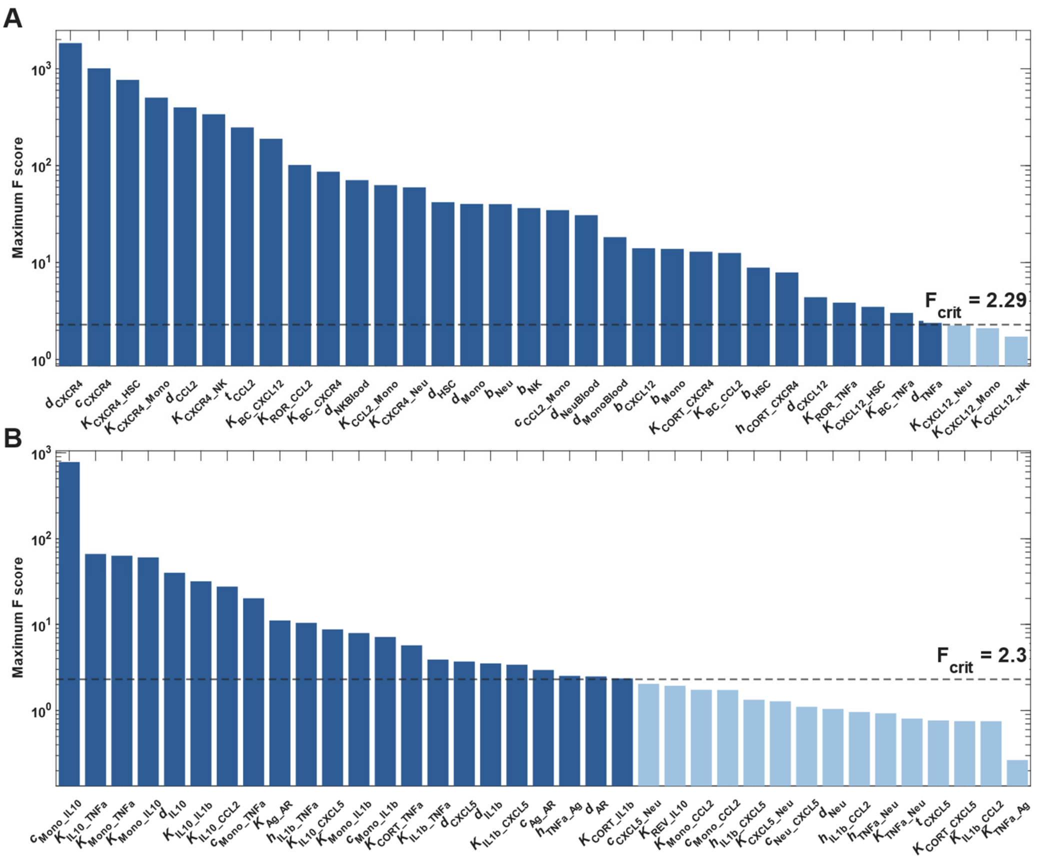

Figure S4. F-normalized profile-based identifiability diagnostics for 71 fitted innate-immune parameters. Parameters were assessed separately for (A) unstimulated basal immune rhythms and (B) stimulation-associated inflammatory responses. For each parameter, the parameter value was fixed sequentially at grid points across its admissible fitting range, while the remaining parameters in the same block were re-optimized. The bar shows the maximum F-normalized profile score obtained across the scanned range. The dashed line indicates the diagnostic threshold. Dark blue bars denote parameters classified as profile-constrained, whereas light blue bars denote weakly constrained and non-identifiable parameters under the current experimental data.

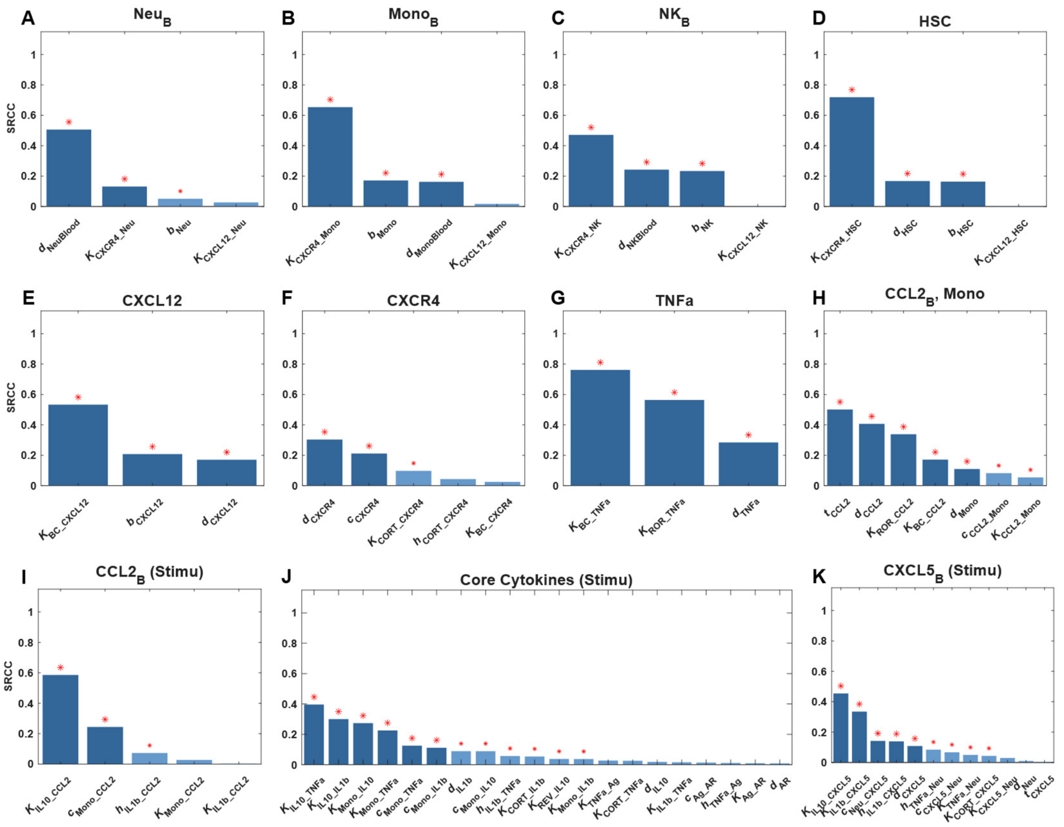

Figure S5. Task-specific SRCC sensitivity screening of fitted immune parameters. Spearman rank correlation coefficients (SRCCs) were calculated between sampled parameter values and the corresponding normalized SSR objective for each fitting task (A–K). Dark blue bars denote sensitive parameters ($|\mathrm{SRCC}| \geq 0.1$), light blue bars with stars denote the ambiguous parameters ($|\mathrm{SRCC}| < 0.1$, $p < 0.05$), and bars without stars denote the insensitive parameters ($p > 0.05$). In the basal cell-trafficking modules (A–D), CXCL12-related thresholds, such as $K_{\mathrm{CXCL12\_Neu}}$, $K_{\mathrm{CXCL12\_Mono}}$, generally showed weaker sensitivity than CXCR4-related parameters. In the CXCR4 module (F), CORT-related parameters showed stronger sensitivity than clock-related parameters. In the stimulated CCL2 module (I), several activation-threshold parameters showed weak sensitivity. The "Core Cytokines" module (J) represents the coupled hub of TNF-α, IL-1β, and IL-10 across all clock conditions; in this module, sensitive, ambiguous, and insensitive parameters accounted for 30%, 30%, and 40% of the fitted parameters, respectively, with insensitivity mainly associated with monocyte-activation-related parameters. In the CXCL5/neutrophil module (K), sensitive, ambiguous, and insensitive parameters

accounted for 42%, 33%, and 25%, respectively, with the latter two groups mainly involving neutrophil-associated terms. SRCC sensitivity screening was used to support mechanistic interpretation of the profile-based identifiability results.

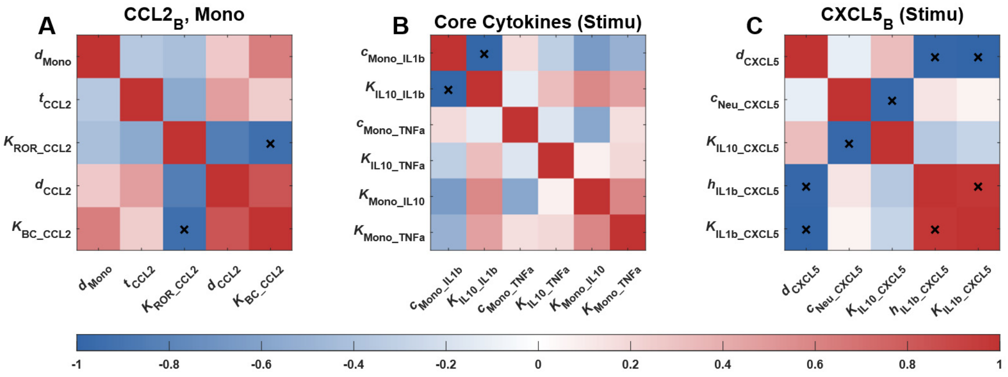

Figure S6. FIM-based local collinearity analysis of immune modules with more than three sensitive parameters. Correlation matrices were derived from Fisher information matrix (FIM) analysis for selected higher-dimensional fitting modules. Black crosses ("×") indicate parameter pairs with absolute correlation coefficients above the predefined collinearity threshold ($|r| > 0.9$). In the CCL2/monocyte module (A), local collinearity was mainly observed between two clock-regulated CCL2 control terms $K_{\mathrm{ROR\_CCL2}}$ and $K_{\mathrm{BC\_CCL2}}$, which have similar function. In the core cytokine module (B), highly correlated pairs $c_{\mathrm{Mono\_IL1b}}$ and $K_{\mathrm{IL10\_IL1b}}$ primarily participate in IL-1β activation and inhibitory regulation, which are mutually antagonistic. In the CXCL5/neutrophil module (C), multiple highly correlated parameter pairs were observed, indicating strong local parameter-compensation relationships.

S2. Mathematical Model Equations

S2.1. Model variables

Table S1. List of variables.

Clock-gene mRNAs are denoted by gene-style names (*Per, Cry, Nr1d, Ror, Bmal1*). For immune variables, the suffix Blood denotes the peripheral blood compartment, and variables without a suffix denote the tissue compartment unless otherwise specified. In the ODE implementation, variables in the peripheral blood compartment are denoted by the suffix Blood (e.g., MonoBlood, IL10Blood), whereas figure labels may use a blood subscript for readability. Unless otherwise specified, immune-cell and cytokine variables without a suffix denote tissue states.

Variable name	Description
Per	Concentration of *Per* mRNA
Cry	Concentration of *Cry* mRNA
Nr1d	Concentration of *Nr1d* mRNA
Ror	Concentration of *Ror* mRNA
Bmal1	Concentration of *Bmal1* mRNA
PER	Concentration of PER proteins
CRY	Concentration of CRY proteins
REV	Concentration of REV proteins
ROR	Concentration of ROR proteins
BMAL1	Concentration of BMAL1 proteins
PER–CRY	Concentration of PER–CRY protein complex

CLOCK–BMAL1	Concentration of CLOCK–BMAL1 protein complex
NeuBlood	Concentration of neutrophils in blood
Neu	Concentration of neutrophils in tissue
MonoBlood	Concentration of circulating monocytes in peripheral blood
Mono	Concentration of monocytes in tissue (lumped monocytes/macrophages)
NKBlood	Concentration of natural killer cells in blood
HSC	Concentration of hematopoietic stem cells in blood
CXCR4	Concentration of CXCR4 on the surface of immune cells
IL10	Concentration of interleukin-10 in tissue
IL10Blood	Concentration of interleukin-10 in blood
IL1b	Concentration of interleukin-1β in tissue
IL1bBlood	Concentration of interleukin-1β in blood
CCL2	Concentration of C-C motif chemokine ligand 2 in tissue
CCL2Blood	Concentration of C-C motif chemokine ligand 2 in blood
CXCL2	Concentration of C-X-C motif chemokine ligand 2 in tissue
TNFa	Concentration of tumor necrosis factor-α in tissue
TNFaBlood	Concentration of tumor necrosis factor-α in blood
CXCL5	Concentration of C-X-C motif chemokine ligand 5 in tissue
CXCL5Blood	Concentration of C-X-C motif chemokine ligand 5 in blood
CXCL12	Concentration of C-X-C motif chemokine ligand 12 in bone marrow
AR	Activation fraction of the tissue Mono population
Ag	External stimulus in tissue (instantiated as LPS in the present simulations)
CORT	Plasma corticosterone concentration

S2.2. Equations of the mathematical model

Equation S1–S12. Circadian mRNAs and proteins

$$\frac{d[Per]}{dt} = -dm_{per}\cdot[Per] + \frac{vmax_{per}\cdot\left(1+fold_{per}\cdot\left(\frac{[\mathrm{BMAL1-CLOCK}]}{Ka_{per-cb}}\right)^{hill_{per-cb}}\right)}{1+\left(\frac{[\mathrm{BMAL1-CLOCK}]}{Ka_{per-cb}}\right)^{hill_{per-cb}}\cdot\left(1+\left(\frac{[\mathrm{PER-CRY}]}{Ki_{per-pc}}\right)^{hill_{per-pc}}\right)} \quad \text{(S1)}$$

$$\frac{d[Cry]}{dt} = -dm_{cry}\cdot[Cry] + \frac{1}{1+\left(\frac{[\mathrm{REV}]}{Ki_{cry-rev}}\right)^{hill_{cry-rev}}} \cdot \frac{vmax_{cry}\cdot\left(1+fold_{cry}\left(\frac{[\mathrm{BMAL1-CLOCK}]}{Ka_{cry-cb}}\right)^{hill_{cry-cb}}\right)^{hill_{cry-cb}}}{1+\left(\frac{[\mathrm{BMAL1-CLOCK}]}{Ka_{cry-cb}}\right)^{hill_{cry-cb}}\cdot\left(1+\left(\frac{[\mathrm{PER-CRY}]}{Ki_{cry-pc}}\right)^{hill_{cry-pc}}\right)^{hill_{cry-pc}}} \quad \text{(S2)}$$

$$\frac{d[Nr1d]}{dt} = -dm_{rev}[Nr1d] + \frac{vmax_{rev}\cdot\left(1+fold_{rev}\cdot\left(\frac{[\mathrm{BMAL1-CLOCK}]}{Ka_{rev-cb}}\right)^{hill_{rev-cb}}\right)^{hill_{rev-cb}}}{1+\left(\frac{[\mathrm{BMAL1-CLOCK}]}{Ka_{rev-cb}}\right)^{hill_{rev-cb}}\cdot\left(1+\left(\frac{[\mathrm{PER-CRY}]}{Ki_{rev-pc}}\right)^{hill_{rev-pc}}\right)} \quad \text{(S3)}$$

$$\frac{d[Ror]}{dt} = -dm_{ror}\cdot[Ror] + \frac{vmax_{ror}\cdot\left(1+fold_{ror}\cdot\left(\frac{[\mathrm{BMAL1-CLOCK}]}{Ka_{ror-cb}}\right)^{hill_{ror-cb}}\right)}{1+\left(\frac{[\mathrm{BMAL1-CLOCK}]}{Ka_{ror-cb}}\right)^{hill_{ror-cb}}\cdot\left(1+\left(\frac{[\mathrm{PER-CRY}]}{Ki_{ror-pc}}\right)^{hill_{ror-pc}}\right)} \quad \text{(S4)}$$

$$\frac{d[Bmal1]}{dt} = -dm_{bmal}[Bmal1] + \frac{vmax_{bmal} \cdot \left(1 + fold_{bmal} \cdot \left(\frac{[\text{ROR}]}{Ka_{bmal-ror}}\right)^{hill_{bmal-ror}}\right)}{1 + \left(\frac{[\text{ROR}]}{Ka_{bmal-ror}}\right)^{hill_{bmal-ror}} + \left(\frac{[\text{REV}]}{Ki_{bmal-rev}}\right)^{hill_{bmal-rev}}} \quad \text{(S5)}$$

$$\frac{d[\text{PER}]}{dt} = -dp_{per} \cdot [\text{PER}] + kp_{per} \cdot [Per] - kass_{pc} \cdot [\text{PER}] \cdot [\text{CRY}] + kdiss_{pc} \cdot [\text{PER} - \text{CRY}] \quad \text{(S6)}$$

$$\frac{d[\text{CRY}]}{dt} = -dp_{cry} \cdot [\text{CRY}] + kp_{cry}[Cry] - kass_{pc} \cdot [\text{PER}] \cdot [\text{CRY}] + kdiss_{pc} \cdot [\text{PER} - \text{CRY}] \quad \text{(S7)}$$

$$\frac{d[\text{REV}]}{dt} = -dp_{rev} \cdot [\text{REV}] + kp_{rev}[Nr1d] \quad \text{(S8)}$$

$$\frac{d[\text{ROR}]}{dt} = -dp_{ror}[\text{ROR}] + kp_{ror}[Ror] \quad \text{(S9)}$$

$$\frac{d[BMAL1]}{dt} = -dp_{bmal} \cdot [\text{BMAL1}] + kp_{bmal} \cdot [Bmal1] - kass_{cb} \cdot [\text{BMAL1}] + kdiss_{cb}[\text{BMAL1} - \text{CLOCK}] \quad \text{(S10)}$$

$$\frac{d[\text{PER} - \text{CRY}]}{dt} = -d_{pc} \cdot [\text{PER} - \text{CRY}] + kass_{pc} \cdot [\text{PER}] \cdot [\text{CRY}] - kdiss_{pc} \cdot [\text{PER} - \text{CRY}] \quad \text{(S11)}$$

$$\frac{d[\text{BMAL1} - \text{CLOCK}]}{dt} = -d_{cb} \cdot [\text{BMAL1} - \text{CLOCK}] + kass_{cb} \cdot [\text{BMAL1}] - kdiss_{cb} \cdot [\text{BMAL1} - \text{CLOCK}] \quad \text{(S12)}$$

Equation S13–S19. Immune cells and receptors

$$\frac{d[\text{NeuBlood}]}{dt} = -d_{\text{NeuBlood}} \cdot [\text{NeuBlood}] + b_{\text{Neu}} \cdot \left(\frac{K^2_{\text{CXCL12\_Neu}}}{K^2_{\text{CXCL12\_Neu}} + [\text{CXCL12}]^2}\right) \cdot \frac{K^3_{\text{CXCR4\_Neu}}}{K^3_{\text{CXCR4\_Neu}} + [\text{CXCR4}]^3} \quad \text{(S13)}$$

$$\frac{d[\text{Neu}]}{dt} = -d_{\text{Neu}} \cdot [\text{Neu}] + \frac{[\text{NeuBlood}]^2}{[\text{NeuBlood}]^2 + K^2_{\text{BT\_Neu}}} \cdot \left(1 + \frac{h_{\text{TNFa\_Neu}} \cdot [\text{TNFa}]}{K_{\text{TNFa\_Neu}} + [\text{TNFa}]}\right) \cdot \left(\frac{c_{\text{CXCL5\_Neu}} \cdot [\text{CXCL5}]^2}{K^2_{\text{CXCL5\_Neu}} + [\text{CXCL5}]^2} + \frac{c_{\text{CXCL2\_Neu}} \cdot [\text{CXCL2}]}{K_{\text{CXCL2\_Neu}} + [\text{CXCL2}]}\right) \quad \text{(S14)}$$

$$\frac{d[\text{MonoBlood}]}{dt} = -d_{\text{MonoBlood}} \cdot [\text{MonoBlood}] + b_{\text{Mono}} \cdot \left(\frac{K^2_{\text{CXCL12\_Mono}}}{K^2_{\text{CXCL12\_Mono}} + [\text{CXCL12}]^2}\right) \cdot \frac{K^4_{\text{CXCR4\_Mono}}}{K^4_{\text{CXCR4\_Mono}} + [\text{CXCR4}]^4} \quad \text{(S15)}$$

$$\frac{d[\text{Mono}]}{dt} = -d_{\text{Mono}} \cdot [\text{Mono}] + \frac{[\text{MonoBlood}]^2}{[\text{MonoBlood}]^2 + K^2_{\text{BT\_Mono}}} \cdot \frac{c_{\text{CCL2\_Mono}} \cdot [\text{CCL2}]^4}{K^4_{\text{CCL2\_Mono}} + [\text{CCL2}]^4} \cdot \left(1 + \frac{h_{\text{TNFa\_Mono}} \cdot [\text{TNFa}]^3}{K^3_{\text{TNFa\_Mono}} + [\text{TNFa}]^3}\right) \quad \text{(S16)}$$

$$\frac{d[\text{NKBlood}]}{dt} = -d_{\text{NKBlood}} \cdot [\text{NKBlood}] + \frac{b_{\text{NK}} \cdot K^3_{\text{CXCL12\_NK}}}{K^3_{\text{CXCL12\_NK}} + [\text{CXCL12}]^3} \cdot \frac{K^3_{\text{CXCR4\_NK}}}{K^3_{\text{CXCR4\_NK}} + [\text{CXCR4}]^3} \quad \text{(S17)}$$

$$\frac{d[\mathrm{HSC}]}{dt} = -d_{\mathrm{HSC}} \cdot [\mathrm{HSC}] + \frac{b_{\mathrm{HSC}} \cdot K^3_{\mathrm{CXCL12\_HSC}}}{K^3_{\mathrm{CXCL12\_HSC}} + [\mathrm{CXCL12}]^3} \cdot \frac{K^4_{\mathrm{CXCR4\_HSC}}}{K^4_{\mathrm{CXCR4\_HSC}} + [\mathrm{CXCR4}]^4} \quad (\mathrm{S18})$$

$$\frac{d[\mathrm{CXCR4}]}{dt} = -d_{\mathrm{CXCR4}}[\mathrm{CXCR4}] + c_{\mathrm{CXCR4}} \cdot \left(1 + \frac{h_{\mathrm{CORT\_CXCR4}} \cdot [\mathrm{CORT}]^4}{K^4_{\mathrm{CORT\_CXCR4}} + [\mathrm{CORT}]^4}\right) \cdot \frac{K^2_{\mathrm{BC\_CXCR4}}}{K^2_{\mathrm{BC\_CXCR4}} + [\mathrm{BMAL1-CLOCK}]^2} \cdot \frac{K_{\mathrm{TNFa\_CXCR4}}}{K_{\mathrm{TNFa\_CXCR4}} + [\mathrm{TNFa}]_{\mathrm{Blood}}} \quad (\mathrm{S19})$$

In this reduced two-compartment formulation, leukocyte recruitment into tissue is represented as an effective influx rather than as a strictly mass-conserved transfer from the circulating pool. Accordingly, circulating leukocyte variables should be interpreted as accessible blood pools sustained by unresolved replenishment from upstream reservoirs.

Equation S20–S34. Immune cytokines, activation state, antigen, and CORT

IL10 in tissue

$$\frac{d[\mathrm{IL10}]}{dt} = -d_{\mathrm{IL10}} \cdot [\mathrm{IL10}] + k_{\mathrm{BT}} \cdot [\mathrm{IL10Blood}]/\mathrm{k_{Blood}} - k_{\mathrm{BT}} \cdot [\mathrm{IL10}] + \left(t_{\mathrm{IL10}} + c_{\mathrm{Mono\_IL10}} \cdot \frac{[\mathrm{Mono}]^2 \cdot AR^2}{[\mathrm{Mono}]^2 \cdot AR^2 + K^2_{\mathrm{Mono\_IL10}}}\right) \cdot \frac{K^3_{\mathrm{REV\_IL10}}}{K^3_{\mathrm{REV\_IL10}} + [\mathrm{REV}]^3} \quad (\mathrm{S20})$$

IL10 in blood

$$\frac{d[\mathrm{IL10Blood}]}{dt} = -d_{\mathrm{IL10Blood}} \cdot [\mathrm{IL10Blood}] - k_{\mathrm{BT}} \cdot [\mathrm{IL10Blood}] + k_{\mathrm{Blood}} \cdot k_{\mathrm{BT}} \cdot [\mathrm{IL10}] \quad (\mathrm{S21})$$

IL1b in tissue

$$\frac{d[\mathrm{IL1b}]}{dt} = -d_{\mathrm{IL1b}} \cdot [\mathrm{IL1b}] + k_{\mathrm{BT}} \cdot [\mathrm{IL1bBlood}]/\mathrm{k_{Blood}} - k_{\mathrm{BT}} \cdot [\mathrm{IL1b}] + \frac{c_{\mathrm{Mono\_IL1b}} \cdot ([\mathrm{Mono}] \cdot [AR])^5}{([\mathrm{Mono}] \cdot [AR])^5 + K^5_{\mathrm{Mono\_IL1b}}} \frac{K^5_{\mathrm{IL10\_IL1b}}}{K^5_{\mathrm{IL10\_IL1b}} + [\mathrm{IL10}]^5} \frac{K_{\mathrm{CORT\_IL1b}}}{K_{\mathrm{CORT\_IL1b}} + [CORT]} \quad (\mathrm{S22})$$

IL1b in blood

$$\frac{d[\mathrm{IL1bBlood}]}{dt} = -d_{\mathrm{IL1bBlood}} \cdot [\mathrm{IL1bBlood}] - k_{\mathrm{BT}} \cdot [\mathrm{IL1bBlood}] + k_{\mathrm{Blood}} \cdot k_{\mathrm{BT}} \cdot [\mathrm{IL1b}] \quad (\mathrm{S23})$$

CCL2 in tissue

$$\frac{d[\mathrm{CCL2}]}{dt} = -d_{\mathrm{CCL2}} \cdot [\mathrm{CCL2}] + k_{\mathrm{BT}} \cdot [\mathrm{CCL2Blood}]/\mathrm{k_{Blood}} - k_{\mathrm{BT}} \cdot [\mathrm{CCL2}] + \left(t_{\mathrm{CCL2}} + c_{\mathrm{Mono\_CCL2}} \cdot \frac{([\mathrm{Mono}] \cdot [AR])^4}{([\mathrm{Mono}] \cdot [AR])^4 + K^4_{\mathrm{Mono\_CCL2}}}\right) \cdot \frac{K_{\mathrm{ROR\_CCL2}}}{K_{\mathrm{ROR\_CCL2}} + [\mathrm{ROR}]} \cdot \frac{K_{\mathrm{BC\_CCL2}}}{K_{\mathrm{BC\_CCL2}} + [\mathrm{BMAL1\text{ - }CLOCK}]} \cdot \frac{K^5_{\mathrm{IL10\_CCL2}}}{K^5_{\mathrm{IL10\_CCL2}} + [\mathrm{IL10}]^5} \cdot \left(1 + h_{\mathrm{IL1b\_CCL2}} \cdot \frac{[\mathrm{IL1b}]}{K_{\mathrm{IL1b\_CCL2}} + [\mathrm{IL1b}]}\right) \quad (\mathrm{S24})$$

CCL2 in blood

$$\frac{d[\text{CCL2Blood}]}{dt} = -d_{\text{CCL2Blood}} \cdot [\text{CCL2Blood}] - k_{\text{BT}} \cdot [\text{CCL2Blood}] + k_{\text{Blood}} \cdot k_{\text{BT}} \cdot [\text{CCL2}] \tag{S25}$$

CXCL2 in tissue

$$\frac{d[\text{CXCL2}]}{dt} = -d_{\text{CXCL2}} \cdot [\text{CXCL2}] + c_{\text{Mono\_CXCL2}} \cdot \frac{([\text{Mono}] \cdot [AR])^2}{([\text{Mono}] \cdot [AR])^2 + K_{\text{Mono\_CXCL2}}^2} \cdot \frac{K_{\text{IL10\_CXCL2}}}{K_{\text{IL10\_CXCL2}} + [\text{IL10}]}\left(1 + \frac{h_{\text{IL1b\_CXCL2}} \cdot [\text{IL1b}]}{K_{\text{IL1b\_CXCL2}} + [\text{IL1b}]}\right) \tag{S26}$$

TNFa in tissue

$$\begin{aligned}\frac{d[\text{TNFa}]}{dt} = & -d_{\text{TNFa}} \cdot [\text{TNFa}] + k_{\text{BT}} \cdot [\text{TNFaBlood}]/\text{k}_{\text{Blood}} - k_{\text{BT}} \cdot [\text{TNFa}] \\ & + \left(t_{\text{TNFa}} + c_{\text{Mono\_TNFa}} \cdot \frac{([\text{Mono}] \cdot [AR])^4}{([\text{Mono}] \cdot [AR])^4 + K_{\text{Mono\_TNFa}}^4}\right) \\ & \cdot \frac{K_{\text{IL10\_TNFa}}^3}{K_{\text{IL10\_TNFa}}^3 + [\text{IL10}]^3} \cdot \left(1 + \frac{h_{\text{IL1b\_TNFa}} \cdot [\text{IL1b}]^3}{K_{\text{IL1b\_TNFa}}^3 + [\text{IL1b}]^3}\right) \\ & \cdot \frac{K_{\text{ROR\_TNFa}}^4}{K_{\text{ROR\_TNFa}}^4 + [\text{ROR}]^4} \cdot \frac{K_{\text{BC\_TNFa}}^3}{K_{\text{BC\_TNFa}}^3 + [\text{BMAL1 - CLOCK}]^3} \cdot \frac{K_{\text{CORT\_TNFa}}^2}{K_{\text{CORT\_TNFa}}^2 + [\text{CORT}]^2}\end{aligned} \tag{S27}$$

$$\frac{d[\text{TNFaBlood}]}{dt} = -d_{\text{TNFaBlood}} \cdot [\text{TNFaBlood}] - k_{\text{BT}} \cdot [\text{TNFaBlood}] + k_{\text{Blood}} \cdot k_{\text{BT}} \cdot [\text{TNFa}] \tag{S28}$$

CXCL5 in tissue

$$\begin{aligned}\frac{d[\text{CXCL5}]}{dt} = & -d_{\text{CXCL5}} \cdot [\text{CXCL5}] + k_{\text{BT}} \cdot [\text{CXCL5Blood}]/\text{k}_{\text{Blood}} - k_{\text{BT}} \cdot [\text{CXCL5}] \\ & + \left(t_{\text{CXCL5}} + c_{\text{Neu\_CXCL5}} \cdot [\text{Neu}]\right) \cdot \frac{K_{\text{IL10\_CXCL5}}^5}{K_{\text{IL10\_CXCL5}}^5 + [\text{IL10}]^5} \\ & \cdot \left(1 + \frac{h_{\text{IL1b\_CXCL5}} \cdot [\text{IL1b}]^5}{K_{\text{IL1b\_CXCL5}}^5 + [\text{IL1b}]^5}\right) \cdot \frac{K_{\text{BC\_CXCL5}}}{K_{\text{BC\_CXCL5}} + [\text{BMAL1 - CLOCK}]} \cdot \frac{K_{\text{CORT\_CXCL5}}^2}{K_{\text{CORT\_CXCL5}}^2 + [\text{CORT}]^2}\end{aligned} \tag{S29}$$

CXCL5 in blood

$$\frac{d[\text{CXCL5Blood}]}{dt} = -d_{\text{CXCL5Blood}} \cdot [\text{CXCL5Blood}] - k_{\text{BT}} \cdot [\text{CXCL5Blood}] + k_{\text{Blood}} \cdot k_{\text{BT}} \cdot [\text{CXCL5}] \tag{S30}$$

CXCL12 in bone marrow

$$\frac{d[\text{CXCL12}]}{dt} = -d_{\text{CXCL12}} \cdot [\text{CXCL12}] + b_{\text{CXCL12}} \cdot \frac{K_{\text{BC\_CXCL12}}^4}{K_{\text{BC\_CXCL12}}^4 + [\text{BMAL1 - CLOCK}]^4} \cdot \frac{K_{\text{TNFa\_CXCL12}}}{K_{\text{TNFa\_CXCL12}} + [\text{TNFaBlood}]} \tag{S31}$$

Activated Rate of Mono

$$\begin{aligned}Eff_{\text{Ag}} = & [\text{Ag}] \cdot \frac{K_{\text{IL10}_{\text{Ag}}}}{K_{\text{IL10}_{\text{Ag}}} + [\text{IL10}]} \cdot \left(1 + h_{\text{TNFa}_{\text{Ag}}} \cdot \frac{[\text{TNFa}]}{K_{\text{TNFa}_{\text{Ag}}} + [\text{TNFa}]}\right) \\ \frac{dAR}{dt} = & -d_{\text{AR}} \cdot AR + c_{\text{Ag\_AR}} \cdot \frac{Eff_{\text{Ag}}^2}{K_{\text{Ag\_AR}}^2 + Eff_{\text{Ag}}^2}\end{aligned} \tag{S32}$$

Decay of antigen in tissue

$$\begin{cases} \dfrac{d[\mathrm{Ag}]}{dt} = 0 & t < t_{\mathrm{stimu}} \\ \dfrac{d[\mathrm{Ag}]}{dt} = -d_{\mathrm{Ag}}[\mathrm{Ag}] & t \geq t_{\mathrm{stimu}} \end{cases} \tag{S33}$$

CORT in blood

$$\begin{cases} \dfrac{d[\mathrm{CORT}]}{dt} = c_{\mathrm{CORT}} \cdot \exp\left(k_{\mathrm{CORT}} \cdot \sin\left(\dfrac{\pi}{12} \cdot (t + \phi_{\mathrm{CORT}} - t_{\mathrm{initial}})\right)\right) - d_{\mathrm{CORT}} \cdot [\mathrm{CORT}] & t < t_{\mathrm{stimu}} \\ \dfrac{d[\mathrm{CORT}]}{dt} = c_{\mathrm{CORT}} \cdot \exp\left(k_{\mathrm{CORT}} \cdot \sin\left(\dfrac{\pi}{12} \cdot (t + \phi_{\mathrm{CORT}} - t_{\mathrm{initial}})\right)\right) & \\ \quad + c_{\mathrm{stimu\_CORT}} \cdot \dfrac{t - t_{\mathrm{stimu}}}{\tau_{\mathrm{CORT\_pulse}}} \cdot e^{1 - \frac{t}{\tau_{\mathrm{CORT\_pulse}}}} - d_{\mathrm{CORT}} \cdot [\mathrm{CORT}] & t \geq t_{\mathrm{stimu}} \end{cases} \tag{S34}$$

Equation S35–S36. Isolated two-variable ODEs for the neutrophil–CXCL5 positive feedback loop

$$\frac{d[Neu]}{dt} = -d_{Neu} \cdot [Neu] + B_{Neu}\left(c_{CXCL5\_Neu} \cdot \frac{[CXCL5]^{n_{CXCL5\_Neu}}}{K_{CXCL5\_Neu}^{n_{CXCL5\_Neu}} + [CXCL5]^{n_{CXCL5\_Neu}}} + C_{CXCL2\_Neu}\right) \tag{S35}$$

$$\frac{d[CXCL5]}{dt} = -d'_{CXCL5} \cdot [CXCL5] + t_{CXCL5} \cdot \frac{C_{CXCL5}}{c_{Neu\_CXCL5}} + C_{CXCL5} \cdot [Neu] \tag{S36}$$

where,

$$C_{\mathrm{CXCL5}} = c_{Neu\_CXCL5} \cdot \frac{K_{BC\_CXCL5}^{n_{BC\_CXCL5}}}{K_{BC\_CXCL5}^{n_{BC\_CXCL5}} + [BMAL1-CLOCK]^{n_{BC\_CXCL5}}} \frac{K_{IL10\_CXCL5}^{n_{IL10\_CXCL5}}}{K_{IL10\_CXCL5}^{n_{IL10\_CXCL5}} + [IL10]^{n_{IL10\_CXCL5}}} \cdot \left(1 + \frac{h_{IL1b\_CXCL5} \cdot [IL1b]^{n_{IL1b\_CXCL5}}}{K_{IL1b\_CXCL5}^{n_{IL1b\_CXCL5}} + [IL1b]^{n_{IL1b\_CXCL5}}}\right) \cdot \frac{K_{CORT\_CXCL5}^{n_{CORT\_CXCL5}}}{K_{CORT\_CXCL5}^{n_{CORT\_CXCL5}} + [CORT]^{n_{CORT\_CXCL5}}},$$

$$B_{Neu} = \left(1 + \frac{h_{TNFa\_Neu} \cdot [TNFa]^{n_{TNFa\_Neu}}}{K_{TNFa\_Neu}^{n_{TNFa\_Neu}} + [TNFa]^{n_{TNFa\_Neu}}}\right) \frac{[NeuBlood]^2}{[NeuBlood]^2 + K_{BT\_Neu}^2}$$

$$C_{CXCL2} = c_{CXCL2\_Neu} \cdot \frac{[CXCL2]}{K_{CXCL2\_Neu} + [CXCL2]}$$

$$d'_{CXCL5} = d_{CXCL5}\left(1 + \frac{K_{BT}}{d_{CXCL5} + K_{BT}}\right)$$

Here, d'_{CXCL5} denotes the effective degradation rate that incorporates exchange of CXCL5 between blood and tissues. During the long-term recovery phase after antigen stimulation, $B_{Neu} \approx 1, C_{CXCL2} \approx 0\ \mathrm{cells/(ml \cdot h)}$. C_{CXCL5} thus serves as an effective control parameter modulated by the circadian clock.

S3. Parameter fitting

In the process of fitting model parameters to experimental data, all numerical optimizations were performed using appropriately normalized sums of squared residuals as objective functions, with genetic algorithms and particle swarm optimization used for parameter search. Since the overall parameter

estimation strategy is summarized in the main text, this section only details the parameter source annotations, technical assumptions, and task-specific objective functions adopted at each fitting stage.

S3.1. Calibration of Disrupted Circadian Clocks and CORT Dynamics

For calibration of the disrupted circadian clocks, the objective function was defined as

$$J_{circadian} = \chi^2_{Per} + \chi^2_{Nr1d} + \chi^2_{Cry} + \chi^2_{Bmal1},$$

with

$$\chi^2_v = \sum_{i=1}^{N} \left[\frac{v_i^{exp} - v^{sim}(t_i)}{\sigma_{v,i}} \right]^2,$$

where $\sigma_{v,i}$ denotes the experimental pointwise error associated with variable v and sampling time t_i. After clock calibration, the CORT module was parameterized phenomenologically under the corresponding circadian backgrounds. The CORT module was represented phenomenologically by a condition-specific basal circadian forcing term and a shared pulse-like acute response after LPS stimulation (Equation S34; Table S4). The degradation rate constant, d_{CORT}, was calculated based on a reported plasma half-life of 15 minutes [3]. The baseline parameters ($c_{\mathrm{CORT}}, k_{\mathrm{CORT}}, \phi_{\mathrm{CORT}}, b_{\mathrm{CORT}}$) were condition-dependent and characterized the amplitude, waveform, phase, and offset of the basal circadian CORT rhythm. In Equation S34, t_{initial} denotes the model onset time relative to Zeitgeber Time 0 (ZT0), and t_{stimu} denotes the time of LPS administration. At the same time, $c_{\mathrm{stimu\_CORT}}$ and $\tau_{\mathrm{CORT\_pulse}}$ governed the LPS-induced surge. Within the modeled LPS dose range, the acute CORT surge was assumed to vary weakly with dose [4]. In addition, available data suggested broadly similar acute CORT responses across circadian conditions [5,6]. Therefore, only the basal rhythmic component was treated as condition-specific. This step ensured that downstream immune fitting was performed under the appropriate circadian and HPA-axis background. For CORT calibration, the objective function was defined as

$$J_{CORT} = \chi^2_{CORT} = \sum_{i=1}^{N} \left[[\mathrm{CORT}]_i^{exp} - [\mathrm{CORT}]^{sim}(t_i) \right]^2.$$

S3.2. Fitting Basal Cell-Trafficking Rhythms and Stimulation-Independent Secretion

Following the fitting sequence summarized in the main text, this subsection provides the prior-range assumptions and task-specific objective functions used for the unstimulated basal immune modules. Most prior parameter sources are listed directly in the supplementary tables and are not repeated here. Many degradation and apoptosis rates were estimated from reported half-life data. However, in several blood-cell modules, effective removal rates were allowed to exceed intrinsic clearance rates, in some cases by up to one order of magnitude, in order to absorb unresolved homing processes and upstream replenishment that were not explicitly modeled. In addition, for blood–tissue exchange, a volume-ratio term was introduced to represent inter-compartment coupling, thereby allowing tissue cytokine concentrations to exceed their circulating counterparts under inflammatory conditions. Consequently, some parameter ranges (such as those of $K_{\mathrm{A\_B}}$ parameters) derived from blood-based measurements, particularly threshold parameters associated with cytokine concentrations, were reinterpreted in the tissue compartment according to this scaling relationship.

Following the sequence described in the main text, steps 1–2 (CXCR4/CXCL12 and circulating cell rhythms) were fitted using standard error-normalized SSR objectives, step 3 (coupled tissue monocyte–blood CCL2 rhythms) used a weighted normalized objective, and step 4 (basal TNF-α) used a peak-

normalized objective. Additionally, because basal circulating TNF-α in healthy mice is extremely low and often approaches or falls below the detection limit in untreated animals, the parameter t_{TNFa} was adjusted such that the unstimulated blood TNF-α concentration was approximately 1 pg/ml as a physiologically plausible reference level [7], while the temporal-profile-related parameters were then fitted against peak-normalized rhythmic TNF-α data measured in rat splenic NK cells [8].

Because steps 1 and 2 targeted relatively weakly coupled single-variable or single-module rhythms, they were fitted using standard error-normalized SSR objectives:

$$J_{1,2} = \chi_v^2 = \sum_{i=1}^{N} \left[\frac{v_i^{exp} - v^{sim}(t_i)}{\sigma_{v,i}} \right]^2,$$

where $v \in \{\text{CXCR4}, \text{CXCL12}, \text{NeuBlood}, \text{MonoBlood}, \text{NKBlood}, \text{HSC}\}$. For step 3, blood CCL2 and tissue monocyte rhythms were optimized jointly because of their strong coupling. A standard error-normalized SSR objective function was used:

$$J_3 = \chi_{\text{Mono}}^2 + \chi_{\text{CCL2Blood}}^2,$$

$$\chi_v^2 = \sum_{i=1}^{N_v} \left[\frac{v_i^{exp} - v^{sim}(t_i)}{\sigma_{v,i}} \right]^2,$$

where N_v denotes the number of experimental data points for variable v. For step 4, the basal TNF-α rhythmic profile was fitted against peak-normalized data. Accordingly, the objective function was written as

$$J_4 = \chi_{\text{TNFa}}^2 = \sum_{i=1}^{N} \frac{\max\left([\text{TNFa}]_i^{exp}\right)}{\sigma_{\text{TNFa},i}} \left[\frac{[\text{TNFa}]_i^{exp}}{\max\left([\text{TNFa}]_i^{exp}\right)} - \frac{[\text{TNFa}]^{sim}(t_i)}{\max\left([\text{TNFa}]^{sim}(t)\right)} \right]^2,$$

S3.3. Fitting Stimulated Inflammatory Responses

As summarized in the main text, the stimulated-response fitting stage consisted of a coupled fit of the core cytokine hub (TNF-α, IL-10, and IL-1β) across normal and disrupted circadian conditions, followed by calibration of the downstream CCL2 and CXCL5 outputs. The detailed composite objective functions used in these two substeps are given below.

These data were combined into the following composite objective function:

$$J_{core} = \frac{\chi_{\text{norm}}^2}{n_{\text{norm}}} + \frac{\chi_{\text{jet}}^2}{n_{\text{jet}}} + \frac{\chi_{\text{aging}}^2}{n_{\text{aging}}},$$

with

$$\chi_{\text{norm}}^2 = \chi_{\text{IL1b,norm}}^2 + \chi_{\text{IL10,norm}}^2 + \chi_{\text{TNFa,norm}}^2,$$

$$\chi_{\text{jet}}^2 = \chi_{\text{IL1b,jet}}^2 + \chi_{\text{IL10,jet}}^2 + \chi_{\text{TNFa,jet}}^2 + \chi_{\text{IL1b,jetNorm}}^2 + \chi_{\text{IL10,jetNorm}}^2 + \chi_{\text{TNFa,jetNorm}}^2,$$

$$\chi_{\text{aging}}^2 = \chi_{\text{IL10,aging}}^2 + \chi_{\text{TNFa,aging}}^2 + \chi_{\text{IL10,agingNorm}}^2 + \chi_{\text{TNFa,agingNorm}}^2.$$

For cytokines other than blood TNF-α, the residual term was defined in the standard error-normalized form,

$$\chi_v^2 = \sum_{i=1}^{N} \left[\frac{v_i^{exp} - v^{sim}(t_i)}{\sigma_{v,i}} \right]^2.$$

For blood TNF-α, because the response spans a broad dynamic range, a logarithmic residual was used:

$$\chi_{\text{TNFaBlood},u}^2 = \sum_{i=1}^{N} \left[\frac{\log\left([\text{TNFaBlood}]_{u,i}^{exp} + 1\right) - \log\left([\text{TNFaBlood}]_u^{sim}(t_i) + 1\right)}{\sigma_{v,i}/\left([\text{TNFaBlood}]_{u,i}^{exp} + 1\right)} \right]^2,$$

where $u \in \{\text{norm, aging, agingNorm}\}$.

Here, $n_{norm}, n_{jet}, n_{aging}$ denote the numbers of data points in the corresponding data groups and were introduced to balance the relative contributions of the three datasets within the composite objective function. Subset jetNorm and agingNorm represent normal-clock datasets extracted from disrupted clock studies [9,10].

The downstream CCL2/CXCL5 calibration used the following objective function:

$$J_{stimu} = \chi_v^2 = \sum_{i=1}^{N} \left[\frac{v_i^{exp} - v^{sim}(t_i)}{\sigma_{v,i}} \right]^2, \quad v \in \{\text{CCL2Blood, CXCL5Blood}\}.$$

These local fitting tasks provided the basis for the subsequent sensitivity screening and parameter-fixing workflow described in Section S4.

S4. Sensitivity Analysis and Parameter Identifiability

We combined local sensitivity analysis, F-normalized profile diagnostics, task-specific SRCC screening, and FIM-based collinearity analysis to evaluate innate-immune parameters. Among these analyses, the F-normalized profile diagnostic was used as the primary criterion for practical identifiability, whereas SRCC and FIM analyses were used as auxiliary evidence to interpret parameter identifiability.

S4.1. Local perturbation sensitivity analysis

To assess how individual immune-related parameters influence the inflammatory response after stimulation, we first performed a local perturbation sensitivity analysis. A total of 92 immune-related parameters were independently increased and decreased by 5%, while all other parameters were kept unchanged. For each perturbed parameter set, the model was simulated under normal circadian conditions following 3 mg/kg LPS stimulation at ZT0. For parameter p_i and output variable v_j, the response amplitude $P_j(p_i)$ was defined as the maximum absolute deviation of v_j from its steady-state average value during the 24 h period after stimulation. The sensitivity score of parameter p_i was calculated as

$$S_i = \sqrt{\sum_j \left[\frac{P_j(p_i + 5\%) - P_j(p_i - 5\%)}{P_j(p_i)} \right]^2}.$$

Thus, a higher sensitivity score indicates that a small local change in the parameter produces a larger change in the simulated peak inflammatory response. As shown in Figure S3, the highest-ranking parameters were mainly associated with monocyte activation, antigen-dependent regulation, and the CCL2/TNF-α/IL-10 cytokine network. These results suggest that upstream monocyte activation and cytokine feedback loops are major determinants of the simulated peak response after LPS stimulation.

S4.2. F-normalized profile-based identifiability diagnostics

To evaluate whether the fitted immune parameters were constrained by the available experimental data, we performed an F-normalized profile-based identifiability diagnostic [11,12]. This analysis was conducted separately for two parameter blocks: the unstimulated basal immune-rhythm block and the stimulation-associated inflammatory-response block. For each block, the analyzed parameters were the fitted parameters listed in Tables S5 and S6 and marked as [F]. The SSR was calculated by summing the

pointwise residuals defined consistently with the fitting objectives described in Section 'S3. Parameter fitting', including error-normalized residuals, peak-normalized residuals for basal TNF-α, and logarithmic residuals for stimulated blood TNF-α where applicable.

For each parameter p_j, its value was fixed sequentially at nine grid points within the admissible fitting range, including the final fitted value. At each fixed value, the remaining fitted parameters in the same block were re-optimized using fminsearch, and the resulting minimized SSR was recorded as $SSR_j(p_j)$. The variation of $SSR_j(p_j)$ across the scanned values of p_j was defined as the profile curve for that parameter.

To reduce the dependence of the diagnostic on the absolute scale of the composite SSR, profile fitting deterioration was F-normalized by the residual variance estimate of the corresponding block [13,14]:

$$F_j(p_j) = \frac{SSR_j(p_j) - SSR_{\mathrm{ref},j}}{SSR_{\mathrm{ref},j}/(N-P)}.$$

Here, $SSR_{\mathrm{ref},j} = \min_{p_j} SSR_j(p_j)$, N denotes the number of experimental data points included in the block, and P denotes the number of fitted parameters profiled in that block. In the present analysis, the unstimulated block contained $P = 34$ fitted parameters and $N = 50$ residual points, whereas the stimulation-associated block contained $P = 37$ fitted parameters and $N = 52$ residual points. Each profile was then summarized by its maximum F-normalized score:

$$F_{j,\max} = \max_{p_j} F_j(p_j).$$

A parameter was classified as constrained by the experimental data, and therefore identifiable in this diagnostic framework, when

$$F_{j,\max} \geq F_{1,N-P,q}$$

Here, $F_{1,N-P,q}$ denotes the q-quantile of the F distribution with 1 and $N-P$ degrees of freedom. Parameters below this threshold were classified as weakly constrained and practically non-identifiable under the current experimental data. In this study, both blocks used a uniform screening threshold of $q = 0.85$. Because the two blocks had different SSR scales and residual degrees of freedom, we summarized profile deterioration using the F-normalized score rather than directly comparing raw ΔSSR values.

As shown in Figure S4, most parameters in the unstimulated block were constrained by the basal rhythm data. Only 3 of 34 parameters were classified as practically non-identifiable (8.8%): $K_{\mathrm{CXCL12,Neu}}$, $K_{\mathrm{CXCL12,NK}}$, and $K_{\mathrm{CXCL12,Mono}}$, all CXCL12-related trafficking thresholds. This may be because CXCL12-related effects were less separable from the stronger CXCR4-dependent constraints on circulating-cell rhythms. By contrast, the stimulation-associated block contained more weakly constrained parameters. Among the 37 fitted parameters in this block, 15 were classified as practically non-identifiable (40.5%). A substantial fraction of these parameters belonged to the CXCL5/neutrophil module. This likely reflects the limited direct experimental constraints on the downstream CXCL5–neutrophil feedback loop, especially because direct stimulation-time-course measurements for neutrophil dynamics were limited, whereas CXCL5 measurements alone cannot fully separate all parameters in this module. More detailed interpretation of these weakly constrained parameters was supported by task-specific SRCC sensitivity screening and FIM-based collinearity analysis, as described below.

S4.3. SRCC sensitivity screening and FIM-based auxiliary interpretation

For each local fitting task, the corresponding fitted parameters (Tables S5–S6) were sampled within their admissible fitting ranges using Latin hypercube sampling. For each sampled parameter set, task-specific normalized SSR values were calculated using the residual definitions described in Section S3. In core cytokine module (Figure S5J, S6B), TNF-α, IL-1β, and IL-10 were screened jointly using datasets from normal, jet-lagged, and aging conditions. Thus, Figure S5 summarizes task-specific SRCC sensitivity based on the corresponding local fitting objectives introduced earlier in "S3. Parameter fitting". Sensitivity was then quantified by the Spearman rank correlation coefficient (SRCC) between each sampled parameter and the corresponding SSR value. Parameters with larger absolute SRCC values were interpreted as having stronger influence on the task-specific fitting objective, whereas parameters with small and non-significant SRCC values were considered weakly influential in that local task. This SRCC analysis was used as a sensitivity screen and as supporting evidence for interpreting the profile-based identifiability results.

To further examine whether weak profile constraints could arise from local parameter compensation, we performed Fisher information matrix (FIM)-based correlation analysis for selected higher-dimensional modules. Figure S6 shows the resulting correlation matrices for the CCL2/monocyte module, the core cytokine module, and the CXCL5/neutrophil module. Parameter pairs with high absolute correlation coefficients indicate local collinearity, suggesting that the effect of changing one parameter may be partly compensated by changes in another parameter under the current experimental constraints. Therefore, the FIM analysis was used to support mechanistic interpretation of weakly constrained parameters identified by the F-normalized profile diagnostics.

Several CXCL12-related trafficking thresholds, including $K_{\mathrm{CXCL12,Neu}}$, $K_{\mathrm{CXCL12,NK}}$, and $K_{\mathrm{CXCL12,Mono}}$, showed low SRCC values in the corresponding local sensitivity analyses. This indicates that these parameters had limited direct influence on the local SSR objectives. Consistently, they were classified as weakly constrained in the profile-based diagnostics. Several weakly constrained parameters ($K_{\mathrm{Mono\_CCL2}}, K_{\mathrm{IL1b\_CCL2}}, K_{\mathrm{TNFa\_Ag}}, K_{\mathrm{CXCL5\_Neu}}, d_{\mathrm{Neu}}, t_{\mathrm{CXCL5}}$) also showed low SRCC values. These results suggest that part of the practical non-identifiability observed in Figure S4 can be attributed to limited local influence on the task-specific fitting objectives.

In the stimulation-associated block, several weakly constrained parameters were located in the CXCL5/neutrophil module. The FIM correlation matrix for this module showed multiple highly correlated parameter pairs, indicating local compensatory relationships among CXCL5 degradation, CXCL5 secretion, and neutrophil-recruitment terms, leading to weak constraints in this module.

S5. Supplementary Parameter Tables

A dash (–) in the unit column denotes a dimensionless parameter. In Tables S5–S6, [F] denotes parameters that were initially estimated by fitting. Labels [E], [R], and [M] denote parameters inferred from experimental data, estimated from physiological ranges, or adopted from previous mathematical models. For [F] parameters, fitting ranges were inferred from the cited references where available, or from physiological constraints described in the corresponding model-calibration steps.

Table S2. Parameters of the normal circadian clock.

All parameters were adopted from Abo et al. [15] and are labelled as [M].

Parameter	Value	Unit	Description
dm_{per}	0.10576	h^{-1}	*Per* mRNA degradation rate constant
dm_{cry}	0.50633	h^{-1}	*Cry* mRNA degradation rate constant
dm_{rev}	0.47914	h^{-1}	*Rev-Erb* mRNA degradation rate constant
dm_{ror}	0.26786	h^{-1}	*Ror* mRNA degradation rate constant
dm_{bmal}	4.6995	h^{-1}	*Bmal1* mRNA degradation rate constant
dp_{per}	0.14989	h^{-1}	PER protein degradation rate constant
dp_{cry}	1.9105	h^{-1}	CRY protein degradation rate constant
dp_{rev}	0.28899	h^{-1}	REV-ERB protein degradation rate constant
dp_{ror}	0.063637	h^{-1}	ROR protein degradation rate constant
dp_{bmal}	0.22534	h^{-1}	BMAL1 protein degradation rate constant
d_{pc}	0.22571	h^{-1}	PER–CRY protein complex degradation rate constant
d_{cb}	0.1709	h^{-1}	CLOCK–BMAL1 protein complex degradation rate constant
$vmax_{per}$	0.83525	$nmol \cdot l^{-1} \cdot h^{-1}$	*Per* mRNA maximal transcription rate
$vmax_{cry}$	1.0418	$nmol \cdot l^{-1} \cdot h^{-1}$	*Cry* mRNA maximal transcription rate
$vmax_{rev}$	0.065746	$nmol \cdot l^{-1} \cdot h^{-1}$	*Rev-Erb* mRNA maximal transcription rate
$vmax_{ror}$	7.2287	$nmol \cdot l^{-1} \cdot h^{-1}$	*Ror* mRNA maximal transcription rate
$vmax_{bmal}$	0.29055	$nmol \cdot l^{-1} \cdot h^{-1}$	*Bmal1* mRNA maximal transcription rate
$fold_{per}$	0.12156	-	Activation ratio of *Per* by CLOCK–BMAL1
$fold_{cry}$	13.828	-	Activation ratio of *Cry* by CLOCK–BMAL1
$fold_{rev}$	130.78	-	Activation ratio of *Rev-Erb* by CLOCK–BMAL1
$fold_{ror}$	0.078569	-	Activation ratio of *Ror* by CLOCK–BMAL1
$fold_{bmal}$	43.306	-	Activation ratio of *Bmal1* by ROR
Ka_{per-cb}	3.3679	nmol/l	Regulation threshold of *Per* by CLOCK–BMAL1
Ki_{per-pc}	0.14178	nmol/l	Regulation threshold of *Per* by PER–CRY
Ka_{cry-cb}	1.5508	nmol/l	Regulation threshold of *Cry* by CLOCK–BMAL1
Ki_{cry-pc}	0.0027556	nmol/l	Regulation threshold of *Cry* by PER–CRY
$Ki_{cry-rev}$	0.64066	nmol/l	Regulation threshold of *Cry* by REV-ERB
Ka_{rev-cb}	0.18454	nmol/l	Regulation threshold of *Rev-Erb* by CLOCK–BMAL1
Ki_{rev-pc}	550.46	nmol/l	Regulation threshold of *Rev-Erb* by PER–CRY

Parameter	Value	Unit	Description
Ka_{ror-cb}	0.56517	nmol/l	Regulation threshold of *Ror* by CLOCK–BMAL1
Ki_{ror-pc}	0.072928	nmol/l	Regulation threshold of *Ror* by PER–CRY
$Ka_{bmal-ror}$	0.076498	nmol/l	Regulation threshold of *Bmal1* by ROR
$Ki_{bmal-rev}$	0.0002375	nmol/l	Regulation threshold of *Bmal1* by REV-ERB
$hill_{per-cb}$	17.025	-	Hill coefficient for regulation of *Per* by CLOCK–BMAL1
$hill_{per-pc}$	22.829	-	Hill coefficient for regulation of *Per* by PER–CRY
$hill_{cry-cb}$	7.4632	-	Hill coefficient for regulation of *Cry* by CLOCK–BMAL1
$hill_{cry-pc}$	2.583	-	Hill coefficient for regulation of *Cry* by PER–CRY
$hill_{cry-rev}$	58.733	-	Hill coefficient for regulation of *Cry* by REV-ERB
$hill_{rev-cb}$	9.3373	-	Hill coefficient for regulation of *Rev-Erb* by CLOCK–BMAL1
$hill_{rev-pc}$	0.95847	-	Hill coefficient for regulation of *Rev-Erb* by PER–CRY
$hill_{ror-cb}$	6.0371	-	Hill coefficient for regulation of *Ror* by CLOCK–BMAL1
$hill_{ror-pc}$	3.2993	-	Hill coefficient for regulation of *Ror* by PER–CRY
$hill_{bmal-ror}$	2.8187	-	Hill coefficient for regulation of *Bmal1* by ROR
$hill_{bmal-rev}$	1.5678	-	Hill coefficient for regulation of *Bmal1* by REV-ERB
kp_{per}	0.77741	h^{-1}	*Per* translation rate
kp_{cry}	0.9308	h^{-1}	*Cry* translation rate
kp_{rev}	0.0004355	h^{-1}	*Rev-Erb* translation rate
kp_{ror}	0.010866	h^{-1}	*Ror* translation rate
kp_{bmal}	0.97306	h^{-1}	*Bmal1* translation rate
$kass_{cb}$	0.0057803	h^{-1}	CLOCK–BMAL1 association rate
$kass_{pc}$	0.15187	$nmol \cdot l^{-1} \cdot h^{-1}$	PER–CRY association rate
$kdiss_{cb}$	0.00022191	h^{-1}	CLOCK–BMAL1 dissociation rate
$kdiss_{pc}$	0.23509	h^{-1}	PER–CRY dissociation rate

Table S3. Parameters modified in disrupted circadian models.

Control values were adopted from Abo et al. [15] ([M]), whereas jet-lag and aging values were estimated by fitting ([F]).

Parameter	Control	Jet-lagged	Aging
$vmax_{per}$	0.8353	0.3826	0.9105
$vmax_{cry}$	1.042	1.194	1.089
$vmax_{rev}$	0.06575	0.02548	0.04977
$vmax_{bmal}$	0.2905	0.2891	0.1459
Ka_{per-cb}	3.368	2.324	2.275
Ka_{cry-cb}	1.551	1.07	1.048
$Ki_{cry-rev}$	0.6407	4.703	0.8231
Ka_{rev-cb}	0.1845	0.1273	0.1247
Ka_{ror-cb}	0.5652	0.39	0.3818
$Ka_{bmal-ror}$	0.0765	0.07982	0.06873
$Ki_{bmal-rev}$	0.00024	0.00027	8.11E-05
$kass_{cb}$	0.00578	0.0061	0.00825
$kass_{pc}$	0.1519	0.5352	1.15
$kdiss_{cb}$	0.00022	3.67E-05	0.00754
$kdiss_{pc}$	0.2351	2.039	2.436
**Source**	[M]	[F]	[F]

Table S4. Parameter values of CORT regulation under different circadian conditions.

Parameter	Unit	Source	Normal Value	Jet-lagged Value	Aging Value
d_{CORT}	h^{-1}	[E]	2.77	2.77	2.77
c_{CORT}	ng/(ml·h)	[F]	0.0061	0.8254	214.2467
k_{CORT}	h^{-1}	[F]	10.5210	4.9697	0.1543
ϕ_{CORT}	h	[F]	18.6330	7.8368	17.5515
b_{CORT}	ng/ml	[F]	110.9830	95.6804	0.3231
$c_{\mathrm{stimu\_CORT}}$	ng/(ml·h)	[F]	2203.01	2203.01	2203.01
$\tau_{\mathrm{CORT\_pulse}}$	h	[E]	1	1	1

Table S5. Parameters of basal cell-trafficking rhythms and stimulation-independent secretion.

Parameter	Value	Unit	Source	Reference
**CXCL12**				
d_{CXCL12}	0.1615	h^{-1}	[F]	Fitted in this study
b_{CXCL12}	1	pg/(mg protein·h)	[F]	Fitted in this study
$K_{\mathrm{BC\_CXCL12}}$	0.0833	nmol/l	[F]	Range derived from [15]

$K_{\mathrm{TNFa\_CXCL12}}$	500	pg/ml	[E]	TNF-α concentration at half-maximal inhibition [16]
**CXCR4**				
d_{CXCR4}	0.55575	h^{-1}	[F]	Derived from complex internalization half-life [17]
c_{CXCR4}	0.65354	MFI/h	[F]	Fitted in this study
$K_{\mathrm{CORT\_CXCR4}}$	120	ng/ml	[F]	Derived from fluctuation [18]
$K_{\mathrm{BC\_CXCR4}}$	0.4	nmol/l	[F]	Derived from fluctuation [15]
$h_{\mathrm{CORT\_CXCR4}}$	1	MFI h^{-1}	[F]	Calculated from peak and basal CXCR4 expression [19]
$K_{\mathrm{TNFa\_CXCR4}}$	100	pg/ml	[E]	Estimated based on [16]
**Neutrophils in blood**				
d_{NeuBlood}	4.566791	h^{-1}	[F]	Range expanded to account for the exclusion of cell homing
b_{Neu}	1.85E+07	cells/(ml·h)	[F]	Estimated from circulating cell counts [19]
$K_{\mathrm{CXCL12\_Neu}}$	5	ng/mg protein	[F]	Derived from steady-state-fluctuation [20]
$K_{\mathrm{CXCR4\_Neu}}$	0.93976	MFI	[F]	Estimated range based on circulating cell counts [19]
$K_{\mathrm{BT\_Neu}}$	3.00E+05	cells/ml	[E]	Set to 50% of the circulating cell count [19]
**Monocytes in blood**				
b_{Mono}	2.42E+06	cells/(ml·h)	[F]	Range derived from circulating cell counts [19]
$K_{\mathrm{CXCL12\_Mono}}$	2	ng/mg protein	[F]	Derived from steady-state-fluctuation [20]
$K_{\mathrm{CXCR4\_Mono}}$	0.49435	MFI	[F]	Derived within steady-state-fluctuation ranges [19]
$d_{\mathrm{MonoBlood}}$	2.3	h^{-1}	[F]	Expanded by one order of magnitude as cell homing is not considered
**NK cells in blood**				
d_{NKBlood}	2	h^{-1}	[F]	Expanded by one order of magnitude as cell homing is not considered
b_{NK}	2.40E+06	cells/(ml·h)	[F]	Estimated range based on circulating cell counts [19]

$K_{\mathrm{CXCL12\_NK}}$	2	ng/mg protein	[F]	Derived within steady-state-fluctuation [20]
$K_{\mathrm{CXCR4\_NK}}$	0.70376	MFI	[F]	Derived within steady-state-fluctuation ranges [19]
**HSCs**				
d_{HSC}	1.84842	h^{-1}	[F]	Expanded by one order of magnitude as cell homing is not considered
b_{HSC}	3628	cells/(ml·h)	[F]	Estimated range based on circulating cell counts [20]
$K_{\mathrm{CXCL12\_HSC}}$	2	ng/mg protein	[F]	Derived within steady-state-fluctuation [20]
$K_{\mathrm{CXCR4\_HSC}}$	0.41836	MFI	[F]	Derived within steady-state-fluctuation ranges [19]
**Monocytes in tissue and CCL2 in blood**				
$c_{\mathrm{CCL2\_Mono}}$	2.80E+05	cells/(ml·h)	[F]	Estimated from d_{Mono} and Mono baseline [21]
$K_{\mathrm{CCL2\_Mono}}$	30	pg/ml	[F]	Derived from steady-state-fluctuation [22]
d_{Mono}	0.59861	h^{-1}	[F]	Fitted in this study
$K_{\mathrm{BT\_Mono}}$	4000	cells/ml	[E]	Set to 50% of the circulating cell count [19]
d_{CCL2}	4.4	h^{-1}	[F]	Estimated based on half-life data [23]
t_{CCL2}	2207	pg/(ml·h)	[F]	Calibrated to steady-state CCL2 concentrations [22]
$K_{\mathrm{ROR\_CCL2}}$	0.13436	nmol/l	[F]	Derived from circadian fluctuation ranges [15]
$K_{\mathrm{BC\_CCL2}}$	0.24036	nmol/l	[F]	Derived from circadian fluctuation ranges [15]
**TNF-α in tissue**				
d_{TNFa}	2.8483	h^{-1}	[F]	Estimated from a half-life of ~10 min [24]
t_{TNFa}	560.71	pg/(ml·h)	[E]	Calibrated to basal murine TNF-α levels (~1 pg/ml) [7]
$K_{\mathrm{ROR\_TNFa}}$	0.45	nmol/l	[F]	Derived from circadian fluctuation ranges [15]
$K_{\mathrm{BC\_TNFa}}$	0.0873	nmol/l	[F]	Derived from circadian fluctuation ranges [15]

Blood-tissue exchange parameters				
k_{Blood}	0.16667	–	[E]	The peritoneal-to-blood volume ratio of mouse [25]
k_{BT}	10	h^{-1}	[M]	[26]

Table S6. Parameters of stimulated inflammatory responses.

Parameter	Value	Unit	Source	Reference
**Core cytokines in blood (Stimulated)**				
$h_{\text{TNFa\_Mono}}$	5	–	[E]	Derived from [27]
$K_{\text{TNFa\_Mono}}$	1.20E+04	pg/ml	[R]	Derived from LPS stimulation fluctuation [2,10]
d_{IL10}	2.77181	h^{-1}	[F]	Estimated based on half-life data [28]
t_{IL10}	1000	pg/(ml·h)	[M]	[15]
$c_{\text{Mono\_IL10}}$	1.10E+05	pg/(ml·h)	[F]	Fitted in this study
$K_{\text{Mono\_IL10}}$	1.20E+06	cells/ml	[F]	Derived from maximal cell density [29]
$K_{\text{REV\_IL10}}$	0.09	nmol/l	[F]	Derived from circadian fluctuation ranges [15]
d_{IL1b}	10	h^{-1}	[F]	Estimated from a half-life of 4 min [30]
$c_{\text{Mono\_IL1b}}$	8.07E+04	pg/(ml·h)	[F]	Estimated from [2]
$K_{\text{Mono\_IL1b}}$	1.92E+06	cells/ml	[F]	Derived from maximal cell density [29]
$K_{\text{IL10\_IL1b}}$	1.40E+04	pg/ml	[F]	Range derived from [2]
$K_{\text{CORT\_IL1b}}$	475	ng/ml	[F]	Derived from CORT inhibition capacity [31]
$c_{\text{Mono\_TNFa}}$	4.65E+08	pg/(cells·h)	[F]	Estimated from [9,10]
$K_{\text{Mono\_TNFa}}$	1.20E+06	cells/ml	[F]	Derived from maximal cell density [29]
$K_{\text{IL10\_TNFa}}$	894.6542	pg/ml	[F]	Range derived from [2]
$h_{\text{IL1b\_TNFa}}$	3	–	[F]	Estimated from [32]
$K_{\text{IL1b\_TNFa}}$	140	pg/ml	[F]	Derived from [2]
$K_{\text{CORT\_TNFa}}$	975	ng/ml	[F]	Range derived from CORT inhibition capacity [33]
d_{AR}	0.1101	h^{-1}	[F]	Range derived from [15]
$c_{\text{Ag\_AR}}$	13.04203	h^{-1}	[F]	Fitted in this study

$K_{\mathrm{Ag\_AR}}$	1.65561	mg/kg	[F]	Upper limit derived from experimental injection dosages [2]
$K_{\mathrm{IL10\_Ag}}$	138	pg/ml	[M]	[15]
$h_{\mathrm{TNFa\_Ag}}$	3.39799	–	[F]	[15]
$K_{\mathrm{TNFa\_Ag}}$	1.02E+04	pg/ml	[F]	Range derived from [2,10]
d_{Ag}	3	h^{-1}	[M]	[15]
**CCL2 in blood (Stimulated)**				
$c_{\mathrm{Mono\_CCL2}}$	2.84E+07	pg/(cells·h)	[F]	Fitted in this study
$K_{\mathrm{Mono\_CCL2}}$	1.20E+06	cells/ml	[F]	Derived from maximal cell density [29]
$K_{\mathrm{IL10\_CCL2}}$	1.50E+04	pg/ml	[F]	Range derived from [2]
$h_{\mathrm{IL1b\_CCL2}}$	0.5	–	[F]	Derived from [32]
$K_{\mathrm{IL1b\_CCL2}}$	500	pg/ml	[F]	Derived from [2]
**CXCL5 in blood (Stimulated)**				
d_{CXCL5}	4.71051	h^{-1}	[F]	Estimated based on half-lives of other cytokines
t_{CXCL5}	6	pg/(ml·h)	[F]	Range estimated from [34]
$c_{\mathrm{Neu\_CXCL5}}$	0.048	pg/(cells·h)	[F]	Fitted in this study
$K_{\mathrm{IL10\_CXCL5}}$	1.34E+04	pg/ml	[F]	Range derived from [2]
$h_{\mathrm{IL1b\_CXCL5}}$	31.14314	–	[F]	Range derived from increase in mouse [35]
$K_{\mathrm{IL1b\_CXCL5}}$	287.6948	pg/ml	[F]	Range derived from [2]
$K_{\mathrm{BC\_CXCL5}}$	0.2	nmol/l	[R]	Derived from circadian fluctuation ranges [15]
$K_{\mathrm{CORT\_CXCL5}}$	1000	ng/ml	[F]	Estimated from CORT inhibition capacity of TNF-α
$h_{\mathrm{TNFa\_Neu}}$	5	–	[F]	Derived from [27]
$K_{\mathrm{TNFa\_Neu}}$	1.20E+04	pg/ml	[F]	Derived from [2]
$c_{\mathrm{CXCL5\_Neu}}$	1.00E+04	cells/(ml·h)	[F]	Estimated from the typical order of Neu in tissue [36]
$K_{\mathrm{CXCL5\_Neu}}$	110	pg/ml	[F]	Derived from [2]
d_{Neu}	0.15	h^{-1}	[F]	Estimated from [37,38]
**CXCL2**				
d_{CXCL2}	0.5	h^{-1}	[E]	Estimated from a CXCL2 half-life of 1–2 h

$c_{\mathrm{Mono\_CXCL2}}$	4.00E+05	pg/(ml·h)	[E]	Calculated from d_{CXCL2} and the typical order of CXCL2 concentration [39]
$K_{\mathrm{Mono\_CXCL2}}$	1.20E+06	cells/ml	[R]	Upper limit derived from maximal cell density [29]
$K_{\mathrm{IL10\_CXCL2}}$	1.40E+04	pg/ml	[R]	Estimated from [2]
$h_{\mathrm{IL1b\_CXCL2}}$	20	–	[E]	Estimated from the >10-fold increase in CXCL2 secretion [40]
$K_{\mathrm{IL1b\_CXCL2}}$	400	pg/ml	[R]	Estimated from [2]
$c_{\mathrm{CXCL2\_Neu}}$	1.00E+04	cells/(ml·h)	[E]	Calculated from the typical order of Neu in tissue [36]
$K_{\mathrm{CXCL2\_Neu}}$	1.00E+04	pg/ml	[R]	Estimated from [39]

Supplementary References

1. Kwon, M.-S.; Seo, Y.-J.; Choi, S.-M.; Won, M.-H.; Lee, J.-K.; Park, S.-H.; Jung, J.-S.; Sim, Y.-B.; Suh, H.-W. The Time-Dependent Effect of Lipopolysaccharide on Kainic Acid-Induced Neuronal Death in Hippocampal CA3 Region: Possible Involvement of Cytokines via Glucocorticoid. *Neuroscience* **2010**, *165*, 1333–1344, doi:10.1016/j.neuroscience.2009.11.060.
2. Lang, V.; Ferencik, S.; Ananthasubramaniam, B.; Kramer, A.; Maier, B. Susceptibility Rhythm to Bacterial Endotoxin in Myeloid Clock-Knockout Mice. *eLife* **2021**, *10*, e62469, doi:10.7554/eLife.62469.
3. Sainio, E.-L.; Lehtola, T.; Roininen, P. Radioimmunoassay of Total and Free Corticosterone in Rat Plasma: Measurement of the Effect of Different Doses of Corticosterone. *Steroids* **1988**, *51*, 609–622, doi:10.1016/0039-128X(88)90056-6.
4. Suzuki, S.; Nakano, K. LPS-Caused Secretion of Corticosterone Is Mediated by Histamine through Histidine Decarboxylase. *Am. J. Physiol. Endocrinol. Metab.* **1986**, *250*, E243–E247, doi:10.1152/ajpendo.1986.250.3.E243.
5. Tateda, K.; Matsumoto, T.; Miyazaki, S.; Yamaguchi, K. Lipopolysaccharide-Induced Lethality and Cytokine Production in Aged Mice. *Infect Immun* **1996**, *64*, 769–774, doi:10.1128/iai.64.3.769-774.1996.
6. Mathias, S.; Schiffelholz, T.; Linthorst, A.C.E.; Pollmächer, T.; Lancel, M. Diurnal Variations in Lipopolysaccharide-Induced Sleep, Sickness Behavior and Changes in Corticosterone Levels in the Rat. *Neuroendocrinology* **2000**, *71*, 375–385, doi:10.1159/000054558.
7. Zager, R.A.; Johnson, A.C.M.; Hanson, S.Y.; Lund, S. Parenteral Iron Compounds Sensitize Mice to Injury-Initiated TNF-Alpha mRNA Production and TNF-Alpha Release. *Am J Physiol Renal Physiol* **2005**, *288*, F290-297, doi:10.1152/ajprenal.00342.2004.
8. Arjona, A.; Sarkar, D.K. Circadian Oscillations of Clock Genes, Cytolytic Factors, and Cytokines in Rat NK Cells. *J. Immunol.* **2005**, *174*, 7618–7624, doi:10.4049/jimmunol.174.12.7618.
9. Castanon-Cervantes, O.; Wu, M.; Ehlen, J.C.; Paul, K.; Gamble, K.L.; Johnson, R.L.; Besing, R.C.; Menaker, M.; Gewirtz, A.T.; Davidson, A.J. Disregulation of Inflammatory Responses by Chronic Circadian Disruption. *J. Immunol.* **2010**, *185*, 5796–5805, doi:10.4049/jimmunol.1001026.
10. Namas, R.A.; Bartels, J.; Hoffman, R.; Barclay, D.; Billiar, T.R.; Zamora, R.; Vodovotz, Y. Combined In Silico, In Vivo, and In Vitro Studies Shed Insights into the Acute Inflammatory Response in Middle-Aged Mice. *PLoS One* **2013**, *8*, e67419, doi:10.1371/journal.pone.0067419.
11. Venzon, D.J.; Moolgavkar, S.H. A Method for Computing Profile-Likelihood-Based Confidence Intervals. *Journal of the Royal Statistical Society: Series C (Applied Statistics)* **1988**, *37*, 87–94, doi:10.2307/2347496.
12. Raue, A.; Kreutz, C.; Maiwald, T.; Bachmann, J.; Schilling, M.; Klingmüller, U.; Timmer, J. Structural and Practical Identifiability Analysis of Partially Observed Dynamical Models by Exploiting the Profile Likelihood. *Bioinformatics* **2009**, *25*, 1923–1929, doi:10.1093/bioinformatics/btp358.

13. Beale, E.M.L. Confidence Regions in Non-Linear Estimation. *Journal of the Royal Statistical Society: Series B (Methodological)* **1960**, *22*, 41–76, doi:10.1111/j.2517-6161.1960.tb00353.x.
14. Donaldson, J.R.; Schnabel, R.B. Computational Experience With Confidence Regions and Confidence Intervals for Nonlinear Least Squares. *Technometrics* **1987**, *29*, 67–82, doi:10.1080/00401706.1987.10488184.
15. Abo, S.M.C.; Layton, A.T. Modeling the Circadian Regulation of the Immune System: Sexually Dimorphic Effects of Shift Work. *PLoS Comput Biol* **2021**, *17*, e1008514, doi:10.1371/journal.pcbi.1008514.
16. Zhang, Q.; Guo, R.; Schwarz, E.M.; Boyce, B.F.; Xing, L. TNF Inhibits Production of Stromal Cell-Derived Factor 1 by Bone Stromal Cells and Increases Osteoclast Precursor Mobilization from Bone Marrow to Peripheral Blood. *Arthritis Res Ther* **2008**, *10*, R37, doi:10.1186/ar2391.
17. Hickey, K.N.; Grassi, S.M.; Caplan, M.R.; Stabenfeldt, S.E. Stromal Cell-Derived Factor-1a Autocrine/Paracrine Signaling Contributes to Spatiotemporal Gradients in the Brain. *Cell Mol Bioeng* **2021**, *14*, 75–87, doi:10.1007/s12195-020-00643-y.
18. Desmet, L.; Thijs, T.; Mas, R.; Verbeke, K.; Depoortere, I. Time-Restricted Feeding in Mice Prevents the Disruption of the Peripheral Circadian Clocks and Its Metabolic Impact during Chronic Jetlag. *Nutrients* **2021**, *13*, 3846, doi:10.3390/nu13113846.
19. He, W.; Holtkamp, S.; Hergenhan, S.M.; Kraus, K.; De Juan, A.; Weber, J.; Bradfield, P.; Grenier, J.M.P.; Pelletier, J.; Druzd, D.; et al. Circadian Expression of Migratory Factors Establishes Lineage-Specific Signatures That Guide the Homing of Leukocyte Subsets to Tissues. *Immunity* **2018**, *49*, 1175-1190.e7, doi:10.1016/j.immuni.2018.10.007.
20. Méndez-Ferrer, S.; Lucas, D.; Battista, M.; Frenette, P.S. Haematopoietic Stem Cell Release Is Regulated by Circadian Oscillations. *Nature* **2008**, *452*, 442–447, doi:10.1038/nature06685.
21. Sundar, I.K.; Ahmad, T.; Yao, H.; Hwang, J.; Gerloff, J.; Lawrence, B.P.; Sellix, M.T.; Rahman, I. Influenza A Virus-Dependent Remodeling of Pulmonary Clock Function in a Mouse Model of COPD. *Sci Rep* **2015**, *5*, 9927, doi:10.1038/srep09927.
22. Minaduola, M.; Aili, A.; Bao, Y.; Peng, Z.; Ge, Q.; Jin, R. The Circadian Clock Sets a Spatial–Temporal Window for Recent Thymic Emigrants. *Immunol Cell Biol* **2022**, *100*, 731–741, doi:10.1111/imcb.12582.
23. Ohtsuki, K.; Hayase, M.; Akashi, K.; Kopiwoda, S.; Strauss, H.W. Detection of Monocyte Chemoattractant Protein-1 Receptor Expression in Experimental Atherosclerotic Lesions: An Autoradiographic Study. *Circulation* **2001**, *104*, 203–208, doi:10.1161/01.CIR.104.2.203.
24. Flick, D.A.; Gifford, G.E. Pharmacokinetics of Murine Tumor Necrosis Factor. *J Immunopharmacol* **1986**, *8*, 89–97, doi:10.3109/08923978609031087.
25. Al Shoyaib, A.; Archie, S.R.; Karamyan, V.T. Intraperitoneal Route of Drug Administration: Should It Be Used in Experimental Animal Studies? *Pharm Res* **2019**, *37*, 12, doi:10.1007/s11095-019-2745-x.
26. Li, J.; Wu, J.; Zhang, J.; Tang, L.; Mei, H.; Hu, Y.; Li, F. A Multicompartment Mathematical Model Based on Host Immunity for Dissecting COVID-19 Heterogeneity. *Heliyon* **2022**, *8*, e09488, doi:10.1016/j.heliyon.2022.e09488.

27. Véliz, L.P.; González, F.G.; Duling, B.R.; Sáez, J.C.; Boric, M.P. Functional Role of Gap Junctions in Cytokine-Induced Leukocyte Adhesion to Endothelium in Vivo. *Am J Physiol Heart Circ Physiol* **2008**, *295*, H1056–H1066, doi:10.1152/ajpheart.00266.2008.
28. Arai, T.; Abe, K.; Matsuoka, H.; Yoshida, M.; Mori, M.; Goya, S.; Kida, H.; Nishino, K.; Osaki, T.; Tachibana, I.; et al. Introduction of the Interleukin-10 Gene into Mice Inhibited Bleomycin-Induced Lung Injury in Vivo. *Am. J. Physiol. Lung Cell. Mol. Physiol.* **2000**, *278*, L914–L922, doi:10.1152/ajplung.2000.278.5.L914.
29. Hermida, M.D.R.; Malta, R.; de S. Santos, M.D.P.C.; dos-Santos, W.L.C. Selecting the Right Gate to Identify Relevant Cells for Your Assay: A Study of Thioglycollate-Elicited Peritoneal Exudate Cells in Mice. *BMC Research Notes* **2017**, *10*, 695, doi:10.1186/s13104-017-3019-5.
30. Klapproth, J.; Castell, J.; Geiger, T.; Andus, T.; Heinrich, P.C. Fate and Biological Action of Human Recombinant Interleukin 1 Beta in the Rat in Vivo. *Eur J Immunol* **1989**, *19*, 1485–1490, doi:10.1002/eji.1830190821.
31. Wu, L.; Zhou, C.; Wu, J.; Chen, S.; Tian, Z.; Du, Q. Corticosterone Inhibits LPS-Induced NLRP3 Inflammasome Priming in Macrophages by Suppressing Xanthine Oxidase. *Mediators Inflamm.* **2020**, *2020*, 6959741, doi:10.1155/2020/6959741.
32. Bonaventura, P.; Lamboux, A.; Albarède, F.; Miossec, P. Differential Effects of TNF-α and IL-1β on the Control of Metal Metabolism and Cadmium-Induced Cell Death in Chronic Inflammation. *PLoS One* **2018**, *13*, e0196285, doi:10.1371/journal.pone.0196285.
33. Lim, H.-Y.; Müller, N.; Herold, M.J.; van den Brandt, J.; Reichardt, H.M. Glucocorticoids Exert Opposing Effects on Macrophage Function Dependent on Their Concentration. *Immunology* **2007**, *122*, 47–53, doi:10.1111/j.1365-2567.2007.02611.x.
34. Koltsova, E.K.; Ley, K. The Mysterious Ways of the Chemokine CXCL5. *Immunity* **2010**, *33*, 7–9, doi:10.1016/j.immuni.2010.07.012.
35. Nunemaker, C.S.; Chung, H.G.; Verrilli, G.M.; Corbin, K.L.; Upadhye, A.; Sharma, P.R. Increased Serum CXCL1 and CXCL5 Are Linked to Obesity, Hyperglycemia, and Impaired Islet Function. *J Endocrinol* **2014**, *222*, 267–276, doi:10.1530/JOE-14-0126.
36. Gibbs, J.; Ince, L.; Matthews, L.; Mei, J.; Bell, T.; Yang, N.; Saer, B.; Begley, N.; Poolman, T.; Pariollaud, M.; et al. An Epithelial Circadian Clock Controls Pulmonary Inflammation and Glucocorticoid Action. *Nat Med* **2014**, *20*, 919–926, doi:10.1038/nm.3599.
37. Zhou, J.; Wang, H.; Ouyang, Q. Mathematical Modeling of Viral Infection and the Immune Response Controlled by the Circadian Clock. *J Biol Phys* **2024**, *50*, 197–214, doi:10.1007/s10867-024-09655-5.
38. Torres, M.; Wang, J.; Yannie, P.J.; Ghosh, S.; Segal, R.A.; Reynolds, A.M. Identifying Important Parameters in the Inflammatory Process with a Mathematical Model of Immune Cell Influx and Macrophage Polarization. *PLoS Comput Biol* **2019**, *15*, e1007172, doi:10.1371/journal.pcbi.1007172.
39. Amano, H.; Yamamoto, H.; Senba, M.; Oishi, K.; Suzuki, S.; Fukushima, K.; Mukaida, N.; Matsushima, K.; Eguchi, K.; Nagatake, T. Impairment of Endotoxin-Induced Macrophage Inflammatory Protein 2 Gene Expression in Alveolar Macrophages in Streptozotocin-Induced Diabetes in Mice. *Infect Immun* **2000**, *68*, 2925–2929, doi:10.1128/iai.68.5.2925-2929.2000.
40. Saijo, Y.; Tanaka, M.; Miki, M.; Usui, K.; Suzuki, T.; Maemondo, M.; Hong, X.; Tazawa, R.; Kikuchi, T.; Matsushima, K.; et al. Proinflammatory Cytokine IL-1 Beta Promotes Tumor Growth of

Lewis Lung Carcinoma by Induction of Angiogenic Factors: In Vivo Analysis of Tumor-Stromal Interaction. *J. Immunol.* **2002**, *169*, 469–475, doi:10.4049/jimmunol.169.1.469.
